# *Her4.3*^+^ radial glial cells maintain the brain vascular network through activation of Wnt signaling

**DOI:** 10.1016/j.jbc.2024.107570

**Published:** 2024-07-15

**Authors:** Pengcheng Wang, Lingfei Luo, Jingying Chen

**Affiliations:** 1 Institute of Developmental Biology and Regenerative Medicine, Southwest University, Chongqing, China; 2 Department of Anaesthesia of Zhongshan Hospital, School of Life Sciences, Fudan University, Shanghai, China

**Keywords:** radial glial cells, cerebral vascular network, blood–brain barrier, Wnt signaling

## Abstract

During vascular development, radial glial cells (RGCs) regulate vascular patterning in the trunk and contribute to the early differentiation of the blood–brain barrier. Ablation of RGCs results in excessive sprouting vessels or the absence of bilateral vertebral arteries. However, interactions of RGCs with later brain vascular networks after pattern formation remain unknown. Here, we generated a her4.3 transgenic line to label RGCs and applied the metronidazole/nitroreductase system to ablate *her4.3*^+^ RGCs. The ablation of *her4.3*^+^ RGCs led to the collapse of the cerebral vascular network, disruption of the blood–brain barrier, and downregulation of Wnt signaling. The inhibition of Wnt signaling resulted in the collapse of cerebral vasculature, similar to that caused by *her4.3*^+^ RGC ablation. The defects in the maintenance of brain vasculature resulting from the absence of *her4.3*^+^ RGCs were partially rescued by the activation of Wnt signaling or overexpression of Wnt7aa or Wnt7bb. Together, our study suggests that *her4.3*^+^ RGCs maintain the cerebral vascular network through Wnt signaling.

Cerebrovascular-related diseases are tied to structural and morphological changes in blood vessels, decreased vascular density, and progressive vascular abnormalities. Thus, targeting cerebral blood vessels is beneficial for treating cerebrovascular-related diseases (1, 2, 3).

The basic vascular network is constructed by vasculogenesis and angiogenesis. Initial blood vessels are formed by the differentiation of mesoderm-derived angioblast, which assemble into a primitive vascular plexus. It recruits periendothelial support cells that encase the endothelial tubes, followed by endothelial cell proliferation and migration, giving off branches or capillaries at different depths to increase blood vessels’ size and complexity, ultimately constructing a highly organized and ramified vascular network (4, 5). In this process, exploring how various cell types and signaling mechanisms regulate the development and stability of vascular networks is a traditional scientific problem.

Radial glial cells (RGCs) are thought to originate from neuroepithelial cells (NECs), which undergo symmetric division to form the neural plate, including the ventricle and subventricular zones. During early neurogenesis, NECs expand the central nervous system through self-renewal and generate neurons or glial cells. As the process continues, NECs lose some epithelial features and gain glial characteristics, ultimately transforming into RGCs (6, 7, 8). The primary function of RGCs is to guide newborn neurons' migration to their destinations in the cerebral cortex through their long radial fibers (9, 10). Additionally, RGCs can differentiate into various cellular types during central nervous system neurogenesis (11, 12, 13). In the central nervous system (CNS) neurogenic niche of adult mammals, RGCs are located in a specialized microenvironment and connected with blood vessels. They terminate their specialized end-feet on the blood vessels (14, 15, 16) and contribute to the neurovascular unit and the blood–brain barrier (BBB) early differentiation (17, 18, 19). Additionally, RGCs can regulate cortical angiogenesis by promoting endothelial cell migration, tube formation, and branching through the secretion of VCAM1 or TGF-β1 Signal (20, 21). When RGCs' division is suppressed, vascular regression can occur, reducing vessel number and density (22).

The canonical Wnt signaling pathway plays a crucial role in regulating vascular development during CNS angiogenesis in zebrafish and mammals, including the growth and specialization of vessels (23). Studies conducted on zebrafish suggested that targeted mutation of *reck* or *gpr124* results in abnormalities in the development of blood vessels in the brain (24, 25). Besides regulating angiogenesis, Wnt signaling also contributes to vascular differentiation in the CNS by regulating the expression of BBB-specific proteins such as claudins, GLUT1, and ATP-binding cassette transporters (26, 27). However, it is still unclear whether the Wnt signaling mediates RGC regulation of the BBB formation and angiogenesis in the central nervous system.

Zebrafish is an excellent model for high-resolution, *in vivo* live imaging of brain vascular angiogenesis and regeneration (28, 29, 30, 31). In zebrafish, RGCs act as negative regulators that control the development of vascular patterns in the trunk. Excessive vessel sprouting can occur if these RGCs are eliminated (32). Additionally, a separate study found that reducing RGCs in the spinal cord can lead to the absence of bilateral vertebral arteries in the trunk (33). However, whether the RGCs play roles in maintaining the cerebral vascular network and contribute to the BBB formation in zebrafish is still unknown.

RGCs exhibit the expression of several molecules that are distinctive of astrocytes, thus attributing to them their glial phenotype, which includes brain lipid-binding protein; astrocyte-specific glutamate transporter GLAST, vimentin, and GFAP (34). Moreover, RGCs share the expression of several markers with neural stem cells (NSCs) or neural epithelial cells, such as NESTIN, SOX2, and HER4.3 (35, 36). *Her4.3*, a homologous gene of *hes5*, has previously been considered for labeling RGCs in zebrafish (37). It belongs to the basic helix-loop-helix (bHLH) gene family and plays an essential role in maintaining the number and diversity of NSCs (38, 39, 40, 41).

Here, we labeled RGCs in zebrafish using *her4.3* and incorporated the metronidazole (MTZ)/nitroreductase (NTR) cell ablation system to specifically eliminate RGCs. The MTZ/NTR system is a genetic technology used to eliminate specific cell types by expressing NTR, a bacterial prodrug converting enzyme. When MTZ, the substrate of NTR, is introduced to the transgenic fish, it eliminates the NTR-expressing RGCs. The ablation of *her4.3*^*+*^ RGCs resulted in the collapse of the cerebral vascular network, disruption of the BBB, and downregulation of Wnt signaling. Similarly, the inhibition of Wnt signaling resulted in the collapse of cerebral vasculature, as caused by *her4.3*^*+*^ RGC ablation. Moreover, the activation of Wnt signaling or overexpression of Wnt7aa or Wnt7bb could partially rescue the defects in the maintenance of brain vasculature due to the absence of *her4.3*^+^ RGCs. Our study suggests that *her4.3*^+^ RGCs maintain the cerebral vascular network through Wnt signaling.

## Results

### RGCs labeled by *her4.3*, connected with brain blood vessels

To investigate the function of RGCs in brain vascular development, we constructed transgenic lines, *Tg(her4.3:eGFPNTR)*^*cq184*^ and *Tg(her4.3:mCherryNTR)*^*cq185*^, enabling label RGCs in zebrafish. Whole-mount *in situ* hybridization confirmed the endogenous expression pattern of *her4.3* is similar to the fluorescence of mCherry and eGFP in our transgenics (Fig. S1, *A* and *B*). In addition, the *her4.3-GFP*^+^ cells displayed long and thin filaments, and when viewed from the dorsal side, these cells had a bipolar shape, this apical-basal radial morphology is a typical characteristic of RGCs (Fig. S1*B*). We also investigated whether *her4.3* labels other neurons or glial cells and found that it does not label *elavl3*^*+*^ neurons, *neurod1*^*+*^ neurons, *sox10*^*+*^ oligodendrocyte precursor cells, and *olig2*^*+*^ oligodendrocytes (Fig. S1, *C*–*F*).

Since RGCs were reported to be labeled by some marker genes, such as *gfap* (42), *nestin* (43), and *sox2* (44), we determined the expression pattern of *gfap* and *her4.3* under the double transgenic background *Tg(her4.3:eGFPNTR; gfap:DsRed)*. From 48 h postfertilization (hpf) to 11 days postfertilization (dpf), *her4.3*^+^ RGCs were found at the midbrain and the intermediate region of the hindbrain and partially overlapped with *gfap*^+^ cells (Fig. S2*A*), the result of flow cytometry indicated *her4.3*^*+*^*gfap*^*+*^ cells account for 27% of all *her4.3*^*+*^ cells (Fig. S2*B*). *Sox2* and *nestin* were also widely used in labeling RGCs. The fluorescence *in situ* hybridization (FISH) combined antibody staining showed a portion of *her4.3*^+^ RGCs expressed *sox2* and *nestin* in the larvae stage (Fig. S2, *C* and *D*). The results indicate that *her4.3* labels a subset of RGCs in zebrafish.

**Figure 1:**
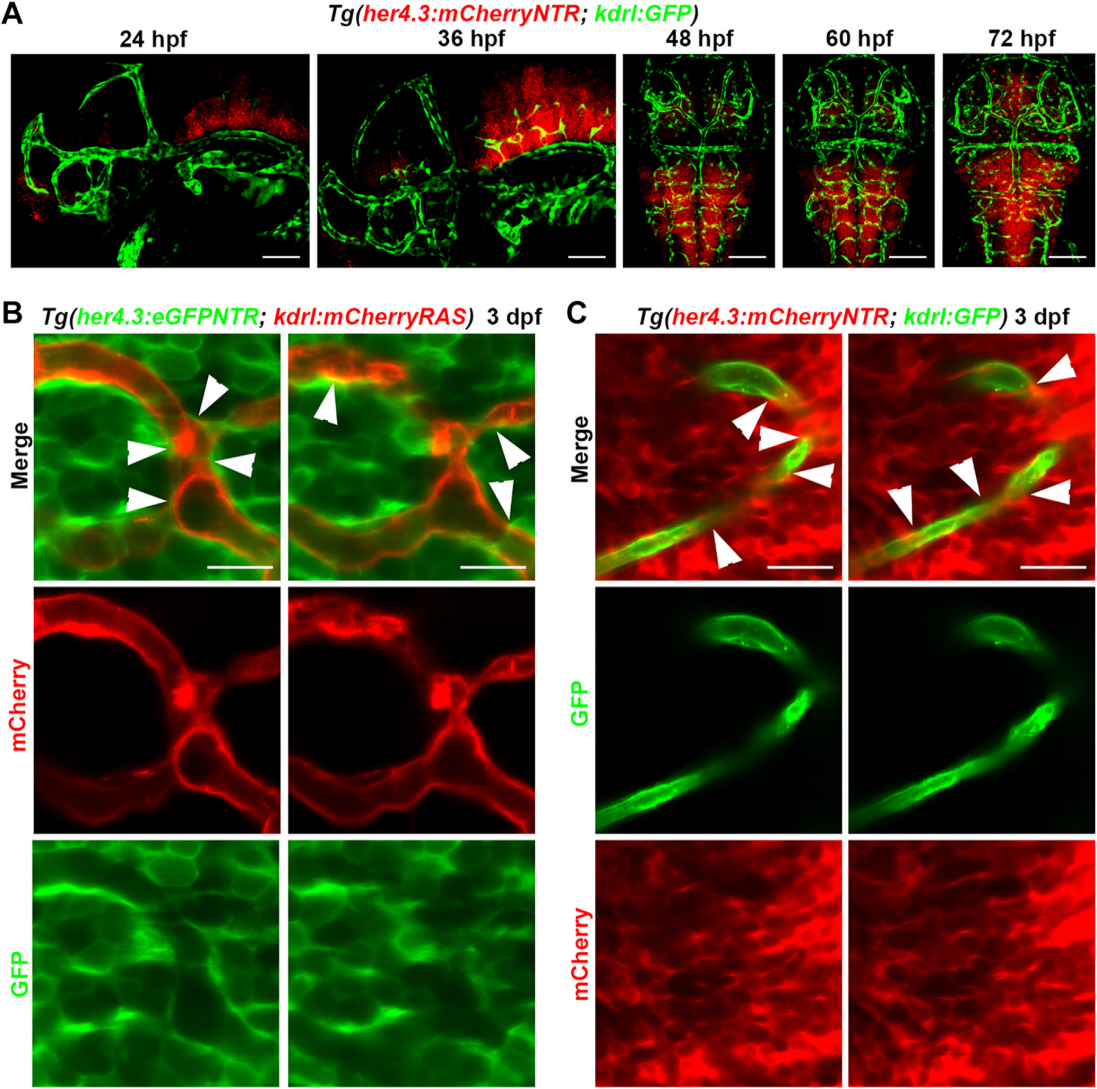
***Her4.3***^**+**^**RGCs connected with cerebral blood vessels.***A*, confocal time-lapse showed *her4.3*^+^ RGCs develop with blood vessels in the brain at 24 hpf, 36 hpf, 48 hpf, 60 hpf, and 72 hpf, under the *Tg(her4.3:mCherryNTR; kdrl:GFP)* transgenic background. Scale bar, 100 μm. *B* and *C*, confocal time-lapse showed blood vessels marked by *kdrl* traversing through radial glial cells labeled by *her4.3* at 3 dpf, under the *Tg(her4.3:eGFPNTR; kdrl:mCherryRAS)* (*B*) or *Tg(her4.3:mCherryNTR; kdrl:GFP)* (*C*) transgenic background. The *white arrow* indicates that *her4.3*^+^ RGCs lie close to blood vessels. Scale bar, 10 μm. dpf, days postfertilization; hpf, hours postfertilization; RGC, radial glial cell.

RGCs were reported to have direct physical connections to blood vessels through cell end-feet processes (16). Tracing the larvae under the transgenic background *Tg(her4.3:mCherryNTR; kdrl:GFP)* from 24 hpf to 72 hpf, we found that *her4.3*^+^ RGCs were widely distributed in the midbrain and hindbrain and developed with the construction of cerebral vascular network at the early stage (Fig. 1*A*). The dynamic single slice imaging permitted the visualization of blood vessels marked by *kdrl* traversing through RGCs in the midbrain region, and *her4.3*^*+*^ RGCs lie close to blood vessels and appeared to ensheath them (Fig. 1, *B* and *C*). These data suggest a physical connection between *her4.3*^+^ RGCs and intracerebral vessels.

### *Her4.3*^*+*^ RGCs are required for cerebral vascular work maintenance

**Figure 2:**
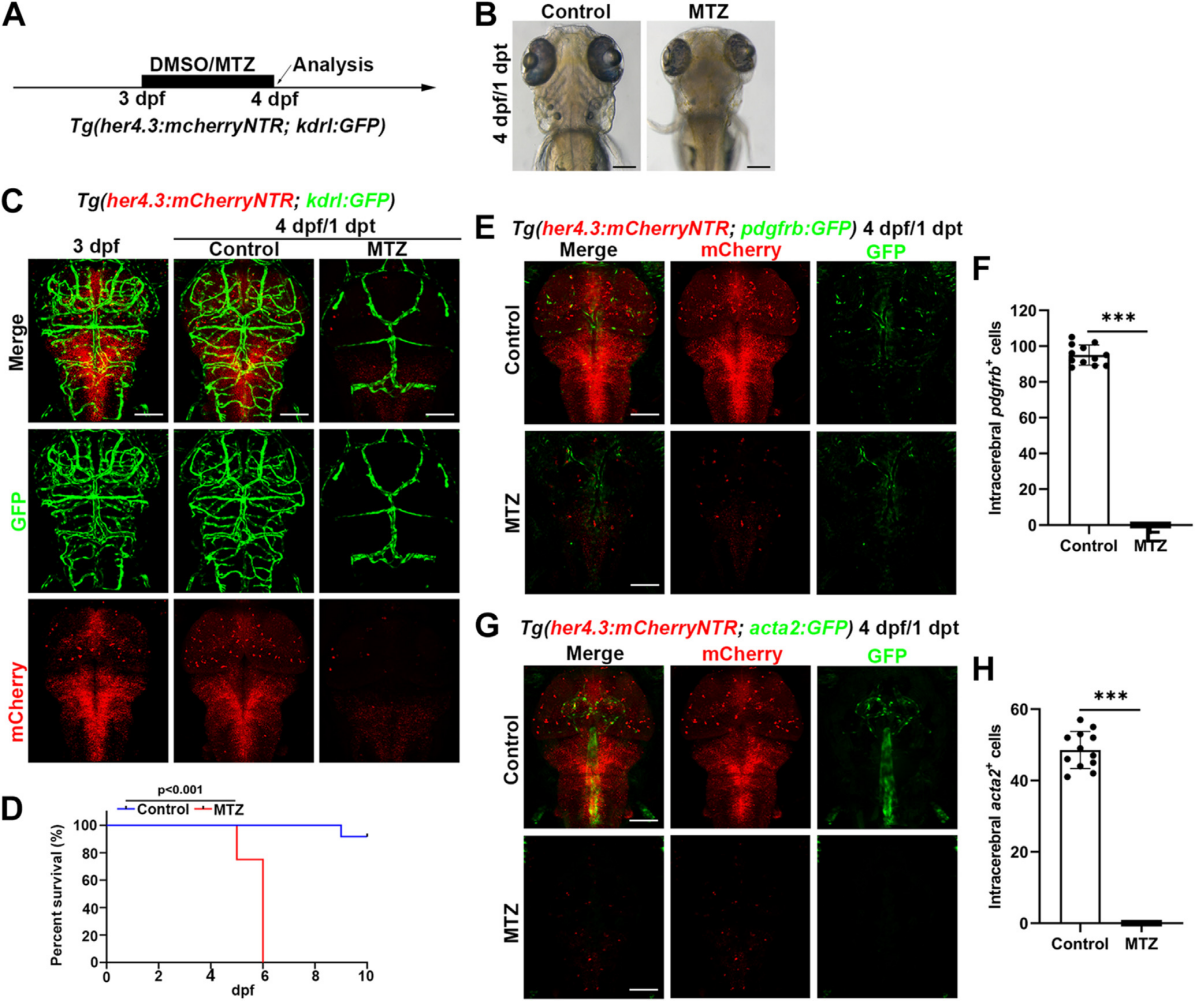
***Her4.3***^**+**^**RGCs are required for the maintenance of the cerebral vascular network.***A*, overview of the time points of DMSO or MTZ treatment. *B*, the brain morphology of larvae in control and MTZ group at 4 dpf/1 dpt (n = 20/20). *C*, confocal images showed intracerebral vessels disappeared from the brain after *her4.3*^+^ RGCs ablation, at 4 dpf/1 dpt, under the *Tg(her4.3:mCherryNTR; kdrl:GFP)* transgenic background (n = 20/20). Control treated with DMSO (n = 20/20). *D*, the survival rate of embryos in the control and MTZ group was monitored from 24 hpf to 10 dpf. n = 12. *p*-value < 0.001. *E*, confocal images showed *pdgfrb*^+^ pericytes (n = 16/16) disappeared from the brain after *her4.3*^+^ RGCs ablation at 4 dpf/1 dpt, under *Tg(her4.3:mCherryNTR; pdgfrb:GFP)* transgenic background. Control treated with DMSO. *F*, quantification of the number of *pdgfrb*^+^ pericytes after DMSO or MTZ treatment at 4 dpf/1 dpt. Control treated with DMSO. n = 12, two-tailed unpaired *t* test, ∗∗∗*p* < 0.001. *G*, confocal images showed *acta2*^+^ smooth muscle cells (n = 16/16) disappeared from the brain after *her4.3*^+^ RGCs ablation at 4 dpf/1 dpt, under *Tg(her4.3:mCherryNTR; acta2:GFP)* transgenic background. Control treated with DMSO. *H*, quantification of the number of *acta2*^+^ smooth muscle cells after DMSO or MTZ treatment at 4 dpf/1 dpt. Control treated with DMSO. n = 12, two-tailed unpaired *t* test, ∗∗∗*p* < 0.001. Scale bar, 100 μm. Data are represented as mean ± SD. dpf, days postfertilization; hpf, hours postfertilization; MTZ, metronidazole; RGC, radial glial cell.

To illustrate the role of *her4.3*^+^ RGCs in regulating cerebral blood vessel development, the MTZ/NTR system was applied to ablate the RGCs. We used 5 mM MTZ to treat the larvae under the *Tg(her4.3:mCherryNTR; kdrl:GFP)* transgenic background at 3 dpf (Fig. 2*A*), when the basic cerebral vascular network was constructed. After treatment of MTZ for 20 h, we removed the MTZ and detected the morphology of the larvae at 4 dpf/1 dpt (days postbeginning treatment). The larvae showed a bending trunk (Fig. S3*A*) and some dark regions in the brain (Fig. 2*B*). Confocal images showed that most *her4.3*^+^ RGCs were ablated in the spinal cord and the brain (Figs. 2*C* and S3*B*). Compared to the blood vessels in the trunk, a large number of intracerebral vessels were absent after *her4.3*^+^ RGCs were eliminated by MTZ, whereas blood vessels on the cranial head were still present, and *gata1a*^+^ blood cells could be observed flowing through the vessels (Figs. 2*C* and S3*B*, Movies S1 and S2). Meanwhile, zebrafish in the MTZ group could not survive later than 6 dpf (Fig. 2*D*). To exclude the side effects of MTZ, we also treated the transgenic lines without the NTR under the same condition, and the *Tg(her4.3:mCherryRAS; kdrl:GFP)* line showed a normal phenotype in brain and body after MTZ treatment (Fig. S4), revealing that the MTZ treatment did not affect the blood vessels and *her4.3*^+^ RGCs in zebrafish.

To investigate the important role of RGCs in maintaining the cerebral vascular network, we conducted experiments where we removed RGCs from zebrafish at the age of 11 dpf and in adulthood. Upon treatment with MTZ, both young and adult zebrafish showed cerebral vascular defects (Fig. S5, *A* and *C*). Specifically, the intracerebral vessels in the sample showed reduced numbers and became fragmented, while there was no difference in the intersegmental vessels compared to the control group (Fig. S5*B*). These findings indicate that the ablation of *her4.3*^+^ RGCs specifically leads to the disruption of the cerebral vascular network, and this phenotype is consistent among both larvae and adult zebrafish.

The terminal deoxynucleotidyl transferase-mediated deoxyuridine triphosphate nick-end labeling (TUNEL) assay was also conducted under a microscope to identify cell apoptosis. Upon visualization, massive TUNEL^+^ cells were observed in the head and found to overlap with *her4.3*^+^ RGCs or *kdrl*^+^ endothelial cells (Fig. S6, *A* and *B*). Quantitative reverse transcriptase PCR (RT-PCR) analysis demonstrated that the expression level of proapoptotic factors *p53* and *bax* were higher in the MTZ group than in the control group, while the antiapoptotic factor *bcl2* was downregulated (Fig. S6*C*), demonstrating that MTZ treatment can induce cell apoptosis. We additionally used propidium iodide (PI) microinjection into the embryos to ascertain whether cell necrosis contributed to this process, imaging that none of the PI^+^ cells could be observed (Fig. S6, *D* and *E*). Collectively, these results demonstrate that MTZ treatment induces cell apoptosis, not involving cell necrosis.

### *Her4.3*^*+*^ RGCs ablation causes BBB dysfunction

In zebrafish, the BBB is made up of capillary endothelial cells, which vascularize the brain. These cells are connected by specialized tight junctions and maintain close contact with surrounding pericytes (45, 46). RGCs resemble astrocytes found in other vertebrates and take over astrocytes to comprise the BBB in zebrafish (47). Thus, we examined the functional impact of the BBB after RGC ablation using various tests.

First, we investigated the effect on other vascular cell units after *her4.3*^+^ RGCs ablation, such as pericytes and smooth muscle cells. We used *Tg(pdgfrb:GFP)* and *Tg(acta2:GFP)* to mark the pericytes and smooth muscle cells in the brain, respectively. When we applied MTZ treatment to ablate the *her4.3*^*+*^ RGCs, we noticed that the *pdgfrb*^+^ pericytes or *acta2*^+^ smooth muscle cells also disappeared distinctly from the brain at 4 dpf/1 dpt (Fig. 2, *E*–*H*). This suggests that the ablation of *her4.3*^+^ RGCs not only blocked vascular endothelial cells but also disrupted other cell units in the BBB.

**Figure 3:**
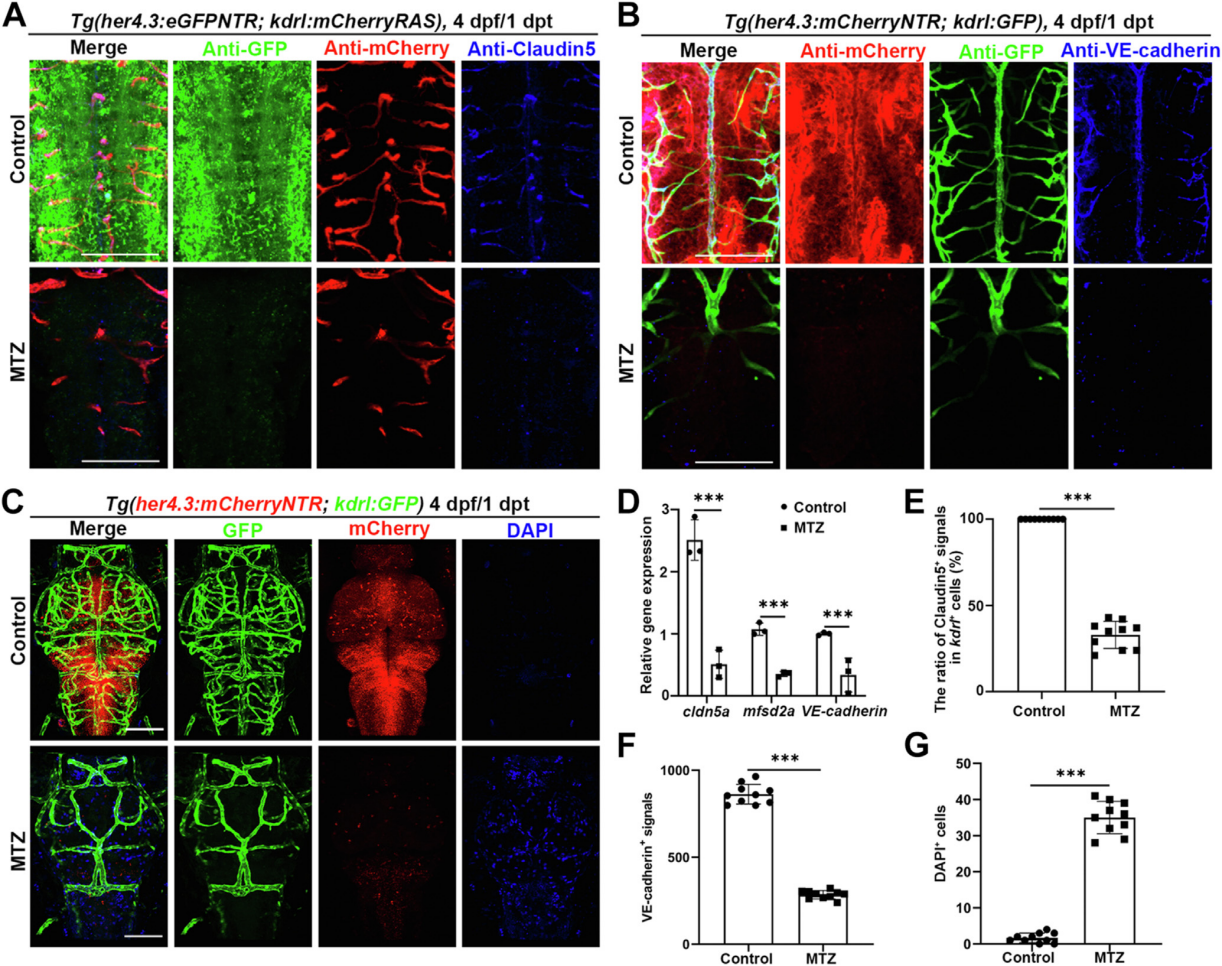
***Her4.3***^**+**^**RGCs ablation disrupts the blood–brain barrier.***A* and *B*, antibody staining for cauldin-5 (n = 9/10) (*A*) or VE-cadherin (n = 10/12) (*B*), under the *Tg(her4.3:eGFPNTR; kdrl:mCherryRAS)* (*A*) or *Tg(her4.3:mCherryNTR; kdrl:GFP)* (*B*) transgenic background after DMSO or MTZ treatment at 4 dpf/1 dpt. Control treated with DMSO. *C*, confocal images showing the result of DAPI injection after DMSO or MTZ treatment under the *Tg(her4.3:mCherryNTR; kdrl:GFP)* transgenic background at 4 dpf/1 dpt. Control treated with DMSO. n = 6/6. *D*, quantitative real-time PCR data showing the relative expression level of *cldn5a*, *mfsd2a* and *VE-cadherin* in the control and MTZ group at 4 dpf/1 dpt. Control treated with DMSO. n = 3 technical replicates. Two-way ANOVA by Sidak’s multiple comparisons test. ∗∗∗*p* < 0.001. *E*, quantification of the ratio of claudin-5^+^ signals in *kdrl*^+^ cells after DMSO or MTZ treatment at 4 dpf/1 dpt. Control treated with DMSO. n = 10, two-tailed unpaired *t* test, ∗∗∗*p* < 0.001. *F*, quantification of the number of VE-cadherin^+^ signals after DMSO or MTZ treatment at 4 dpf/1 dpt. Control treated with DMSO. n = 10, two-tailed unpaired *t* test, ∗∗∗*p* < 0.001. *G*, quantification of the number of DAPI^+^ cells after DMSO or MTZ treatment at 4 dpf/1 dpt. Control treated with DMSO. n = 10, two-tailed unpaired *t* test, ∗∗∗*p* < 0.001. Scale bar, 100 μm. Data are represented as mean ± SD. DAPI, 4ʹ,6-diamidino-2-phenylindole; dpf, days postfertilization; hpf, hours postfertilization; MTZ, metronidazole; RGC, radial glial cell.

We then examined the expression level of some factors known to be associated with BBB function, including claudin-5, VE-cadherin, and mfsd2a. We found that these factors all downregulated distinctively after MTZ treatment (Fig. 3, *A*, *B* and *D*–*F*). To investigate the impairment of BBB function after *her4.3*^+^ RGCs ablation, the leakage tracer, 4ʹ,6-diamidino-2-phenylindole (DAPI), was administered through microinjection into the circulatory vascular system in *Tg(her4.3:mCherryNTR; kdrl:GFP)* at 4 dpf/1 dpt. We discovered a significantly greater accumulation of DAPI-positive cell nuclei in the brain of the MTZ treatment group, in comparison to the control (Fig. 3, *C* and *G*), indicating that BBB permeability was disrupted after *her4.3*^+^ RGCs ablation. Based on the above results, *her4.3*^+^ RGCs play a crucial role in the functioning and maintenance of the BBB's integrity.

### *Her4.3*^*+*^ RGCs regulate the cerebral vascular network through Wnt signaling

**Figure 4:**
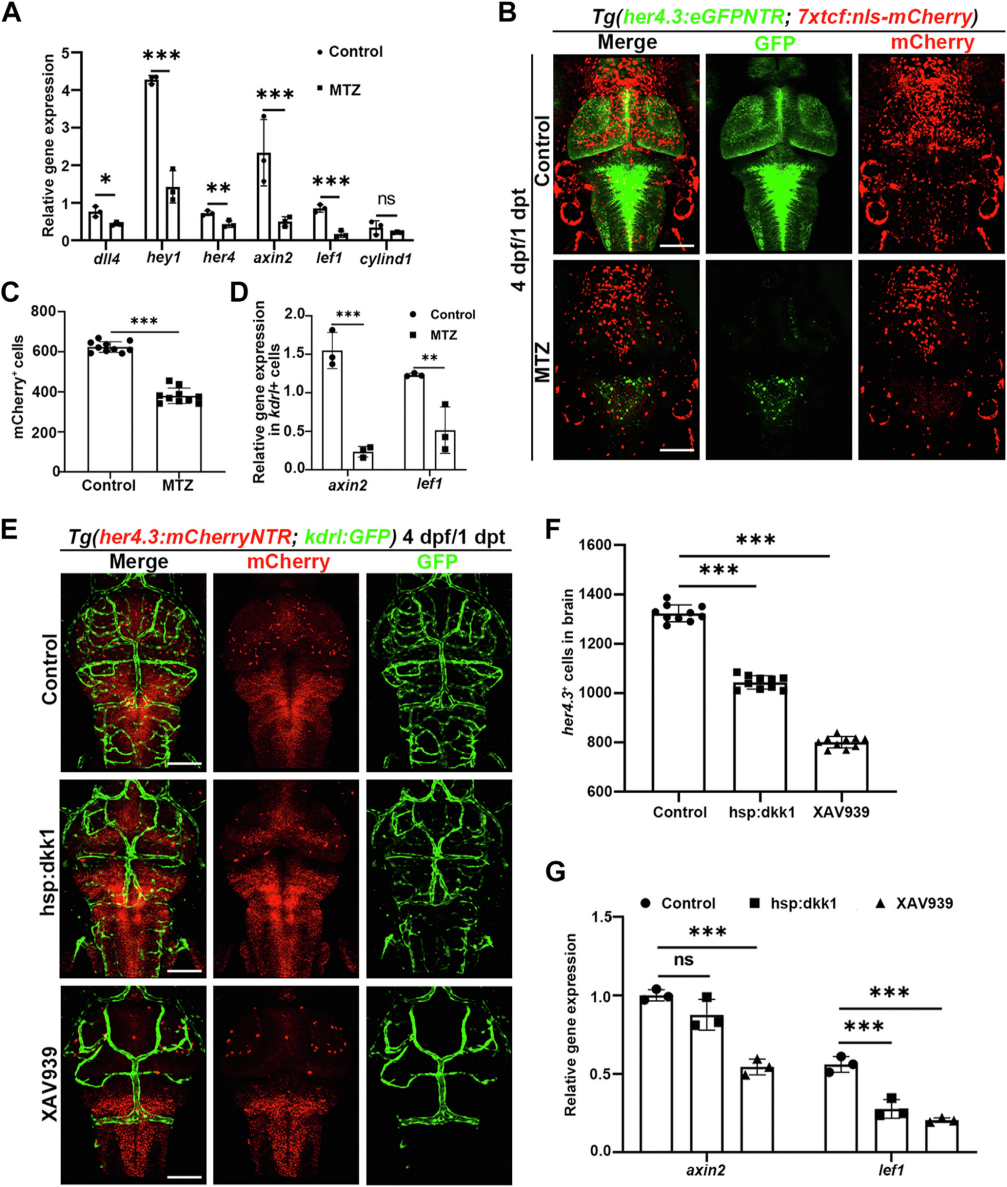
***Her4.3***^**+**^**RGCs regulate the cerebral vascular network through Wnt signaling.***A*, quantitative real-time PCR data showing the relative expression levels of target genes associated with Notch and Wnt in the control and MTZ group at 4 dpf/1 dpt. *dll4*, *hey1*, *her4* for Notch and *axin2*, *lef1*, *cylind1* for Wnt. Control treated with DMSO. n = 3 technical replicates. Two-way ANOVA by Sidak’s multiple comparisons test, ns, no significance, ∗*p* < 0.05 (*p* = 0.016462), ∗∗*p* < 0.01 (*p* = 0.009228), ∗∗∗*p* < 0.001. *B*, confocal images showed the Wnt signaling response to *her4.3*^+^ RGCs ablation, using Wnt reporter line *Tg(7xtcf:nls-mCherry)* at 4 dpf/1 dpt. Control treated with DMSO. n = 6/6. *C*, quantification of the number of mCherry^+^ cells after DMSO or MTZ treatment. Control treated with DMSO. n = 10, two-tailed unpaired *t* test, ∗∗∗*p* < 0.001. *D*, quantitative real-time PCR data showing the relative expression levels of *axin2* and *lef1* after DMSO or MTZ treatment. Control treated with DMSO. n = 3 technical replicates. Two-way ANOVA by Sidak’s multiple comparisons test, ∗∗*p* < 0.01 (*p* = 0.009865) and ∗∗∗*p* < 0.001. *E*, confocal images showed the response of blood vessels and *her4.3*^+^ RGCs after different treatments at 4 dpf/1 dpt. Control treated with DMSO and heat shock (n = 6/6), other groups treated with XAV939 (n = 12/12), or heated to overexpressing dkk1 (n = 8/12). *F*, quantification of the number of *her4.3*^+^ cells after different treatments at 4 dpf/1 dpt. Control treated with DMSO and heat shock, other groups treated with XAV939, or heated to overexpressing dkk1. n = 10, two-tailed unpaired *t* test, ∗∗∗*p* < 0.001. *G*, quantitative real-time PCR data showing the relative expression levels of *axin2* and *lef1* after different treatments at 4 dpf/1 dpt. Control treated with DMSO and heat shock, other groups treated with XAV939, or heated to overexpressing dkk1. n = 3 technical replicates. Two-way ANOVA by Sidak’s multiple comparisons test, ns, no significance, ∗∗∗*p* < 0.001. Scale bar, 100 μm. Data are represented as mean ± SD. dpf, days postfertilization; hpf, hours postfertilization; MTZ, metronidazole; RGC, radial glial cell.

Traditional signaling pathways that regulate cerebral vascular development include Notch and Wnt, and we sought to identify signaling pathways that regulate vascular endothelial cells’ response to *her4.3*^+^ RGCs ablation. Given that *her4.3* belongs to the bHLH gene family, dependent on Notch signaling to regulate NSCs (39), Wnt signaling has previously been indicated to promote NSC proliferation and differentiation in the optic tectum of zebrafish (48). RT-PCR technique was used to check some target genes associated with these signaling pathways, *dll4*, *hey1*, and *her4* for the Notch signaling and *axin2*, *lef1*, and *cyclind1* for the Wnt signaling, and we found that these target genes all significantly downregulated after *her4.3*^+^ RGCs ablation to varying degrees (Fig. 4*A*). Next, we will identify which signaling pathway plays a major role in this process after *her4.3*^+^ RGCs ablation.

*Tg(tp1:nls-mCherry)*, a reporter line of Notch signaling, along with RT-PCR results showing downregulation of *hey1* and *her4* in *tp1*^+^ cells, confirmed that Notch signaling was downregulated after *her4.3*^+^ RGCs ablation (Fig. S7, *A*–*C*). To further investigate the role of Notch signaling in our model, we used *Tg(hsp70l:DNMAML:GFP)* and DAPT to inhibit Notch signaling during the brain vascular development, and the effectiveness of Notch inhibition was confirmed by RT-PCR (Fig. S7*E*). After heat shock and DAPT treatment, we observed the number of *her4.3*^+^ RGCs slightly increased, especially in the hindbrain, while intracerebral vessels did not show any discernible differences (Fig. S7, *D* and *F*). Moreover, the embryo that Notch signaling was blocked presented with a curved trunk, similar to the body morphology after *her4.3*^+^ RGCs ablation, and blood vessels in the trunk remained normal at 4 dpf (Fig. S7*G*). These observations imply that the abnormal trunk resulting from the elimination of *her4.3*^+^ RGCs may be linked to Notch signaling inhibition. However, the specific cerebral vascular defects resulting from *her4.3* elimination are independent of Notch.

The Wnt pathway reporter line *Tg(7xtcf:nls-mCherry)* showed that mCherry-positive cells had dramatically declined upon *her4.3*^+^ RGCs ablation (Fig. 4, *B* and *C*), and the expression level of *axin2* and *lef1* was declined by sorting *kdrl*^+^ endothelial cells for RT-PCR (Fig. 4*D*), suggesting the inactivation of the Wnt signaling after *her4.3*^+^ RGCs ablation and its potential involvement in maintaining the cerebral vascular network. To investigate the relationship between Notch and Wnt signaling, DAPT was used to treat larvae with the *Tg(her4.3:eGFPNTR; 7xtcf:nls-mCherry)* transgenic background, the results indicated that inhibition of Notch signaling did not have a significant effect on the number of mCherry^+^ cells (Fig. S7, *H* and *I*), confirming that there are no crosstalk between Notch and Wnt signaling in our model.

To further investigate the functional significance of Wnt singling in maintaining the cerebral vascular network, a *Tg(hsp70l:dkk1)* transgenic line with an inducible dickkopf, a secreted inhibitor of Wnt signaling, was applied. The *Tg(her4.3:mCherryNTR; kdrl:GFP; hsp70l:dkk1)* triple-transgenic larvae embryos were heat-shocked for 35 min per 6 h after 72 hpf. At 4 dpf, after heat-shock, *her4.3*^+^ RGCs and intracerebral vessels were reduced, in comparison to the control that blood vessels maintained normal (Fig. 4, *E* and *F*). To validate these results, a chemical inhibitor of Wnt signaling, XAV939 (49), was used to treat embryos from 3 dpf to 4 dpf. Compared with the control, XAV939 treatment led to a decrease of *her4.3*^+^ RGCs and the absence of a vast majority of the intracerebral vessels in the brain, similar to the phenotype caused by MTZ treatment (Figs. 4, *E*–*F* and 2*C*). The effect of Wnt inhibition was confirmed by RT-PCR that the expression level of *axin2* and *lef1* declined (Fig. 4*G*). These results suggest that inhibition of Wnt signaling influences maintaining the cerebral vascular network. The sample that Wnt signaling was blocked by overexpressing dkk1 or XAV939 treatment was also assessed by TUNEL assay, and large amounts of TUNEL^+^ cells were observed in the brain and overlapped with *her4.3*^+^ RGCs or *kdrl*^+^ endothelial cells (Fig. S8), indicating that Wnt signaling inhibition induced cell apoptosis.

**Figure 5:**
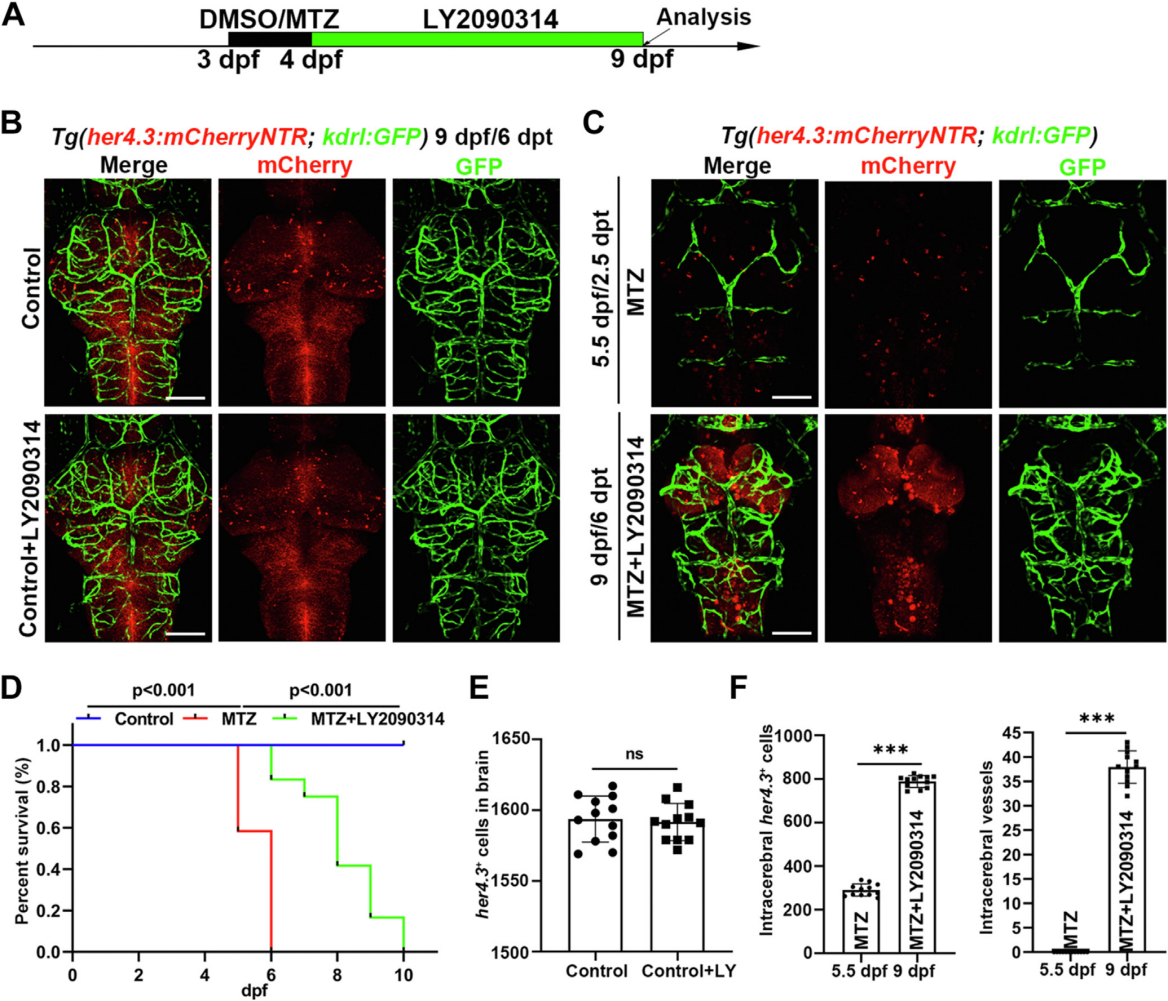
**Wnt signaling activation partially rescues the collapse of the cerebrovascular network caused by *her4.3***^**+**^**RGCs elimination.***A*, overview of the time points of DMSO or MTZ and LY2090314 treatment. *B*, confocal images showed the embryo after DMSO treatment at 9 dpf/6 dpt. The upper group was treated with DMSO, and the bottom group was treated with DMSO + LY2090314. n = 10/10. *C*, confocal images showed the embryo after different treatments. The upper group was treated with MTZ at 5.5 dpf/2.5 dpt, and the bottom group was treated with MTZ + LY2090314 at 9 dpf/6 dpt. n = 7/10. *D*, the survival rate of embryos after different treatments was monitored from 24 hpf to 10 dpf. Control treated with DMSO, sample treated with MTZ, or MTZ + LY2090314. n = 12. *p*-value < 0.001. *E*, quantification of the number of *her4.3*^+^ cells in brain at 9 dpf/6 dpt. Control + LY, Control + LY2090314. n = 12, two-tailed unpaired *t* test. ns, no significance. *F*, quantification of the number of *her4.3*^+^ cells and vessels in brain at 5.5 dpf and 9 dpf. n = 12, two-tailed unpaired *t* test, ∗∗∗*p* < 0.001. Scale bar, 100 μm. Data are represented as mean ± SD. dpf, days postfertilization; hpf, hours postfertilization; MTZ, metronidazole; RGC, radial glial cell.

Next, we sought to elucidate whether the defect of cerebral vascular could be rescued by Wnt signaling activation. LY2090314, a glycogen synthase kinase 3 inhibitor, can activate Wnt signaling effectively (50). We used LY2090314 to treat the larvae from 4 dpf when MTZ was removed (Fig. 5*A*). RT-PCR results indicated *axin2* and *lef1* were upregulated after LY2090314 treatment (Fig. S9*B*). Although at the beginning of LY2090314 treatment (4.5 dpf/1.5 dpt), no obvious effect could be seen compared to the MTZ group (Fig. S9*A*). However, at 9 dpf/6 dpt, confocal live images showed that some intracerebral vessels reappeared in the brain of the sample treated with LY2090314, along with *her4.3*^+^ RGCs increased, these embryos were capable of surviving to a later stage. Whereas the embryo in the MTZ group showed missing intracerebral vessels at 5.5 dpf/2.5 dpt, and all died at 6 dpf (Fig. 5, *B*–*F*).

In summary, the present results imply that activating Wnt signaling could increase the survival rate of embryos after MTZ and partially rescue the collapse of the cerebrovascular network caused by *her4.3*^+^ RGCs ablation, revealing a role of Wnt signaling during the process of RGCs regulating intracerebral vessels.

### *Wnt7aa* and *wnt7bb* downregulation phenocopies the disruption of the cerebrovascular network caused by her4.3^+^ RGCs ablation

**Figure 6:**
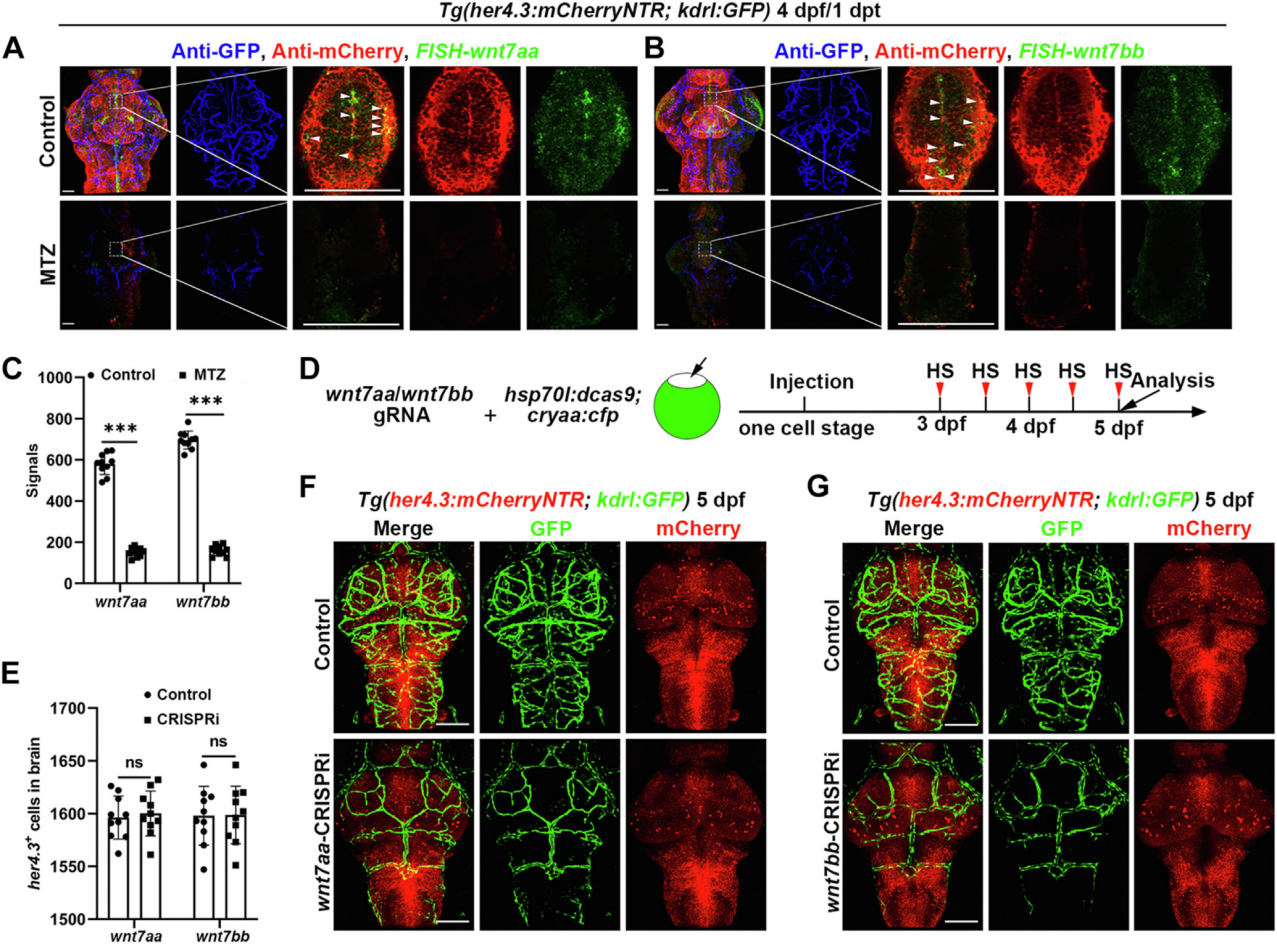
***Wnt7aa* and *wnt7bb* downregulation phenocopy the disruption of the cerebrovascular network caused by *her4.3***^**+**^**RGCs ablation.***A* and *B,* fluorescent *in situ* hybridization (FISH) and antibody staining results showed the expression of *wnt7aa* (n = 10/10) (*A*) or *wnt7bb* (n = 8/10) (*B*) after DMSO or MTZ treatment at 4 dpf/1 dpt, under the *Tg(her4.3:mCherryNTR; kdrl:GFP)* transgenic background. Control treated with DMSO. Higher magnification 2D images of the *white framed areas* are displayed, the *white arrow* indicates the overlap region. Scale bar, 50 μm. *C*, quantification of the number of *wnt7aa*^*+*^ or *wnt7bb*^*+*^ signals after DMSO or MTZ treatment. Control treated with DMSO. n = 10, two-tailed unpaired *t* test. ∗∗∗*p* < 0.001. *D*, overview of the knockdown strategy for *wnt7aa* or *wnt7bb* and the timepoint of heat-shock (*red arrow*). *E*, quantification of the number of *her4.3*^+^ cells in brain at 5 dpf after CRISPRi. n = 10, two-tailed unpaired *t* test. ns, no significance. *F* and *G,* confocal images showed the cerebral vascular pattern after heat shock at 5 dpf, under the *Tg(her4.3:mCherryNTR; kdrl:GFP)* transgenic background. *wnt7aa*-CRISPRi (n = 12/12) (F) and *wnt7bb*-CRISPRi (n = 12/12) (*G*). Control treated with DMSO and heat shock (n = 6/6). Scale bar, 100 μm. Data are represented as mean ± SD. MTZ, metronidazole; dpf, days postfertilization; hpf, hours postfertilization; RGC, radial glial cell.

To identify how Wnt signaling impacts the effect of *her4.3*^+^ RGCs on blood vessels, we used the RT-PCR technique to check some Wnt ligands genes, including *wnt1, wnt2bb, wnt3a, wnt4b, wnt5b, wnt6b, wnt7aa*, and *wnt7bb*, and the results showed that *wnt7aa* and *wnt7bb* were significantly downregulated after MTZ treatment (Fig. S10*A*). In zebrafish, there are two copies of each of the *wnt7a* and *wnt7b* genes, which are further divided into *wnt7aa*, *wnt7ab*, *wnt7ba*, and *wnt7bb*. And Wnt7 plays important roles in regulating mammalian CNS angiogenesis and maintaining the BBB (51, 52, 53). Through FISH and combined antibody staining, we observed that there is a high degree of colocalization between the signals of *wnt7aa* or *wnt7bb* with *her4.3*^+^ RGCs (Fig. 6, *A* and *B*). However, the signals of *wnt7ab* or *wnt7ba* exhibited limited overlap regions with *her4.3*^+^ RGCs (Fig. S10, *B* and *C*). Moreover, the expression levels of these four genes were significantly reduced to varying degrees after *her4.3*^+^ RGCs ablation (Figs. 6*C* and S10*D*). Additionally, *wnt7aa* and *wnt7bb* were upregulated after LY2090314 treatment (Fig. S10*E*), suggesting that *wnt7aa* and *wnt7bb* may play a potential regulatory role in the interaction between blood vessels and *her4.3*^+^ RGCs. To identify the receptors of Wnt7 expressed in blood vessels, we focused on Reck and Gpr124; they are reported to play roles in brain angiogenesis (53). After sorting *kdrl*^+^ cerebral vascular endothelial cells for RT-PCR detection, we found a significant downregulation of *reck* and *gpr124* in blood vessels after *her4.3*^+^ RGCs ablation, then *reck* and *gpr124* were upregulated after LY2090314 treatment (Fig. S10, *F* and *G*). Antibody staining further confirmed that the Wnt reporter line significantly reduced in claudin-5^+^ endothelial cells after *her4.3*^+^ RGCs elimination by MTZ, suggesting a significant role of Wnt signaling in RGC regulating blood vascular network maintenance (Fig. S11, *A* and *B*).

Due to the downregulation of Wnt signaling after RGCs elimination, we next explore whether the inhibition of Wnt signaling can phenocopy the blood vascular defect caused by *her4.3*^+^ RGCs ablation. We first utilized clustered regularly interspaced short palindromic repeat interference (CRISPRi) to knock down Wnt ligands by transcriptional inhibition (54), which had previously been successfully used in the zebrafish (55). At the one-cell stage of the embryo under the transgenic background *Tg(her4.3:mCherryNTR; kdrl:GFP)*, *hsp70l:dcas9; cryaa:cfp* plasmid and sgRNAs of *wnt7aa, wnt7ab, wnt7ba*, or *wnt7bb* were coinjected, and the larvae were then heat-shocked with a time frame of 6 h per day from 3 dpf and analyzed at 5 dpf (Figs. 6*D* and S12*A*). Our results showed that the *wnt7ab* and *wnt7ba* CRISPRi groups did not exhibit any obvious defective phenotype and had a normal cerebrovascular network (Fig. S12, *B* and *C*). However, the *wnt7aa* and *wnt7bb* CRISPRi group showed a significant reduction in intracerebral vessels, similar to the phenotype observed after *her4.3*^+^ RGCs ablation by MTZ treatment, and no significant changes were observed in the number of RGCs (Fig. 6, *E*–*G*). Moreover, *wnt7aa* or *wnt7bb* CRISPRi did not exhibit any conspicuous impact on blood vessels in the trunk (Fig. S12, *D* and *E*). Taken together, these findings strongly support the hypothesis that *wnt7aa* and *wnt7bb* play a crucial role in the effect of *her4.3*^+^ RGCs on blood vessels.

**Figure 7:**
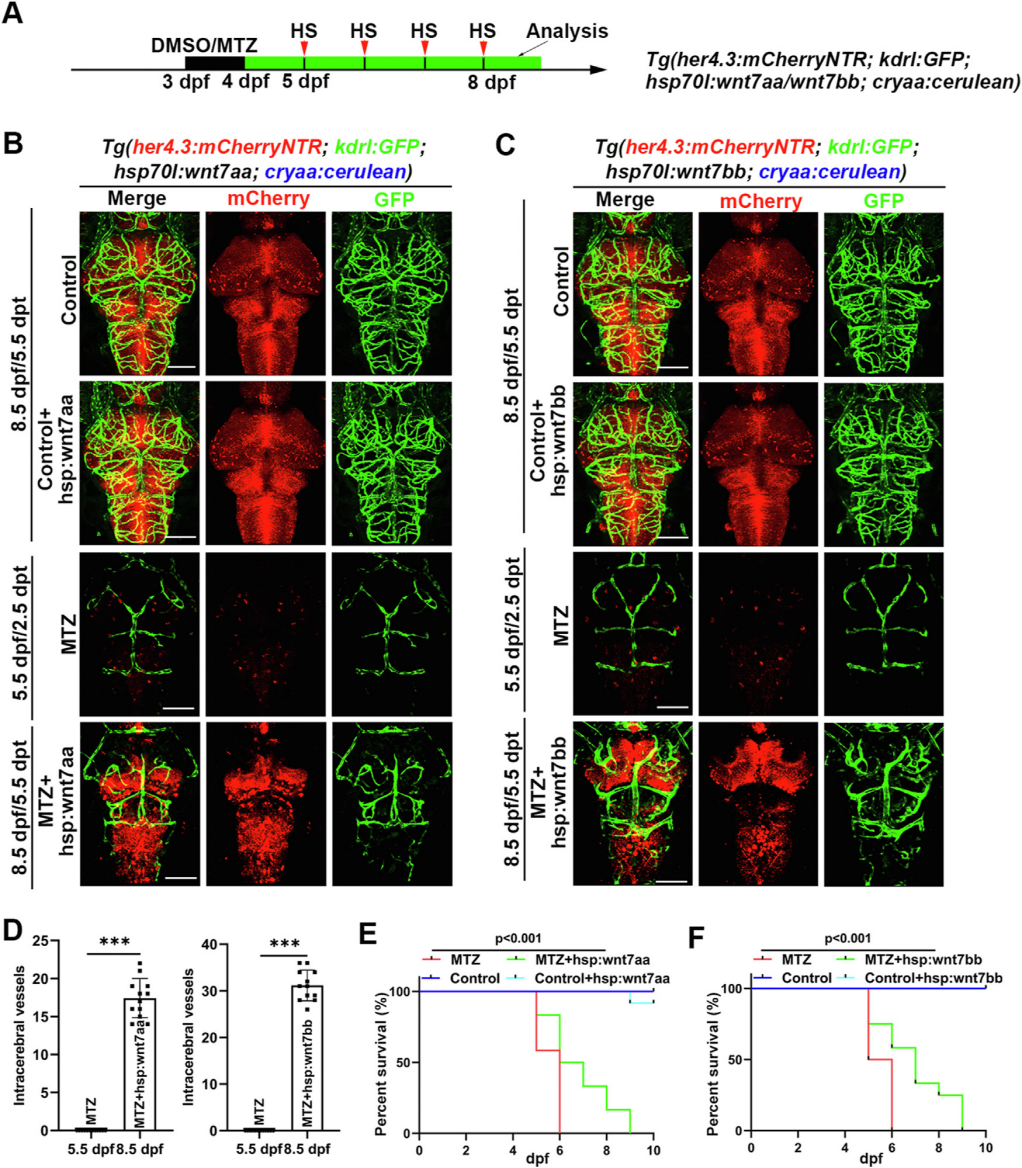
***Wnt7aa* or *wnt7bb* overexpression partially rescues the collapse of the cerebrovascular network caused by *her4.3***^**+**^**RGCs elimination.***A*, overview of the timepoints of DMSO/MTZ and heat-shock (*red arrow*), under *Tg(her4.3:mCherryNTR; kdrl:GFP; hsp70l:wnt7aa; cryaa:cerulean) or Tg(her4.3:mCherryNTR; kdrl:GFP; hsp70l:wnt7bb; cryaa:cerulean)* transgenic background. *B* and *C,* confocal images showed the phenotype in the brain after different treatments at 8.5 dpf/5.5 dpt, under the *Tg(her4.3:mCherryNTR; kdrl:GFP; hsp70l:wnt7aa; cryaa:cerulean)* (n = 10/12) (*B*) *or Tg(her4.3:mCherryNTR; kdrl:GFP; hsp70l:wnt7bb; cryaa:cerulean)* (n = 8/12) (*C*) transgenic background. Control treated with DMSO. The third line showed phenotype in the brain after MTZ treatment at 5.5 dpf/2.5 dpt (n = 12/12). *D*, quantification of the number of intracerebral vessels after MTZ treatment at 5.5 dpf and 8.5 dpf. n = 12, two-tailed unpaired *t* test. ∗∗∗*p* < 0.001. *E* and *F,* the survival rate of embryos after different treatments was monitored from 24 hpf to 10 dpf. Control treated with DMSO or DMSO + hsp:wnt7aa (*E*)/hsp:wnt7bb (*F*), other groups treated with MTZ or MTZ + hsp:wnt7aa (*E*)/hsp:wnt7bb (*F*). n = 12. *p*-value < 0.001. Scale bar, 100 μm. Data are represented as mean ± SD. dpf, days postfertilization; hpf, hours postfertilization; MTZ, metronidazole; RGC, radial glial cell.

Moreover, we created two plasmids named *hsp70l:wnt7aa; cryaa:cerulean* and *hsp70l:wnt7bb; cryaa:cerulean* in order to produce two mosaic overexpression transgenic line. These plasmids were then individually injected into *Tg(her4.3:mCherryNTR; kdrl:GFP)* embryos, which were later treated with MTZ (Fig. 7*A*). After MTZ treatment, the larvae were heat-shocked with a time frame of 24 h per day from 4 dpf/1 dpt to 8.5 dpf/5.5 dpt, and the impact on the cerebral vascular defective phenotype was assessed. At 8.5 dpf/5.5 dpt, larvae overexpressing *wnt7aa* or *wnt7bb* showed a recovery phenotype, with some blood vessels reappearing in the brain, compared to the absence of blood vessels in the MTZ group at 5.5 dpf/2.5 dpt (Fig. 7, *B*–*D*). Additionally, the survival rate of larvae following MTZ treatment was significantly improved by the overexpression of either *wnt7aa* or *wnt7bb* (Fig. 7, *E* and *F*). Overall, based on the above results, it can be inferred that *wnt7aa* and *wnt7bb* are crucial in regulating the effect of *her4.3*^+^ RGCs on blood vessels.

**Figure 8:**
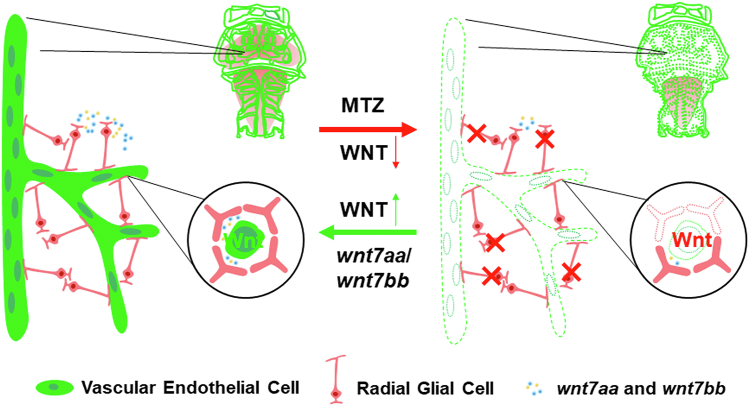
**The model of radial glial cells regulates the cerebral vascular network through Wnt signaling.** In this model, radial glial cells are widely distributed around zebrafish brain and have contact with blood vessels. After MTZ treatment, most *her4.3*^+^ RGCs were ablated, leading to the collapse of the cerebral vascular network and intracerebral vessels disappeared from the brain. Moreover, most *her4.3*^+^ RGCs ablation also disrupted the blood–brain barrier. This process is accompanied by the downregulation of Wnt signaling, and Wnt signaling activation or the stimulation of *wnt7aa* or *wnt7bb* can rescue the defective phenotype. MTZ, metronidazole; RGC, radial glial cell.

In conclusion, our findings indicate that most *her4.3*^+^ RGCs ablation results in the collapse of the cerebral vascular network specifically, compromising the integrity of the BBB. This phenotype appears to be associated with the disruption of Wnt signaling, activating Wnt signaling, and overexpressing *wnt7aa*/*wnt7bb* can partially rescue the defects in the maintenance of brain vasculature (Fig. 8).

## Discussion

Traditionally, single-cell transcriptomics and RNA sequencing have found many genes are highly expressed on RGCs, including *her4.3*, *gfap*, *nestin*, *sox2*, and *blbp* (8, 56). By comparing the expression levels of her4.3 with other markers, we found that *her4.3* overlaps with *gfap*, *nestin*, and *sox2*, indicating that it labels a subset of RGCs in zebrafish. In addition, previous studies have shown that in addition to RGCs, *gfap*, *nestin* and *sox2* label other cell types in zebrafish, including NECs, neural progenitor cells, neural crest cells and intermediate progenitor cells (43, 44, 57). Indeed, as we found that *her4.3* doesn't completely overlap with *gfap*, *sox2*, *nestin*, these results confirmed that there are no absolutely specific markers to label RGCs, due to the complex distribution of RGCs in the zebrafish brain. Similar to previous finding (37), we also observed ectopic expression of *her4.3* in the diencephalon. It is possible that our *her4.*3 transgenic line may label cells other than RGCs, which could be attributed to the 3.4k promoter sequence. However, we did not find any overlap between *her4.3*^*+*^ RGCs and other neurons or glial cells, in either the diencephalon or the hindbrain. In future studies, a new *her4.3* transgenic line will be constructed using a knock-in strategy, allowing for more accurate representation of the distribution of *her4.3*^*+*^ cells.

During CNS angiogenesis, few studies have shown that RGCs regulate blood vessels. Previous research has found that a decrease in RGCs in the spinal cord results in a complete absence of bilateral vertebral arteries (33). Another study demonstrated that the ablation of RGCs leads to excessive sprouting of intersegmental vessels (32), resulting in an increase in vessel density in the trunk. However, our model yielded different results. Ablation of *her4.3*^+^ RGCs caused abnormal body morphology, but the blood vessels in the trunk remained normal with no obvious phenotype, such as vessel loss or overgrowth. In addition, the deletion of a gene that was essential for DNA replication to block RGC division led to the reduction of vessel density and branch tips, as well as hemorrhage in the midbrain (22). In our model, most intracerebral vessels disappeared from the brain after *her4.3*^+^ RGCs ablation at the larvae stage. This phenotype appeared to be more severe than previously reported, although it did not result in hemorrhage. Our experiments on adult fish also showed that the defect caused by *her4.3*^+^ RGCs ablation was conserved. The fish died off in relatively shorter times and could not survive after *her4.3*^+^ RGCs ablation, indicating that RGCs are essential for their survival. However, the main cause of death requires further investigation. Additionally, *her4.3*^+^ RGC ablation resulted in the collapse of the cerebral vascular network. It is worth exploring whether this is due to the large number of *her4.3*^+^ cells ablated by MTZ treatment, causing the vascular network to lose support or a decrease in key factors secreted by RGCs. Further investigation into other potential reasons would be interesting.

Undoubtedly, the interaction between RGCs and endothelial cells is an important cell-cell communication mechanism that promotes the establishment of the BBB. *In vitro* studies have shown that co-culturing brain microendothelial cells with differentiated neural progenitor cells can induce a BBB phenotype (19, 58), by increasing the expression of tight junctions and permeability coefficients. RGCs also contributed to neuron-vascular coupling and early BBB differentiation by promoting neurogenesis and maintaining integrity (59). After the ablation of *her4.3*^+^ RGCs, most endothelial cells, pericytes, and smooth muscle cells disappeared from the brain, which are essential components of the BBB. Normal BBB function also requires junctional proteins, claudins, occludins, and some transporters (45, 60). As our results showed, the expression level of junctional protein, claudin-5 and VE-cadherin, and other transporters were reduced in the MTZ group. This, combined with the tracer analysis, suggests that *her4.3*^+^ RGCs ablation was detrimental to the function and integrity of the BBB. However, the mechanism responsible for the maintenance of the BBB by RGCs and its’ breaking down after RGCs ablation is unknown and would be interesting for future investigations.

*Her4.3*, a member of the bHLH gene family, plays a crucial role in the function of RGCs by relying on Notch signaling. It also regulates the proliferation and differentiation of RGCs (39, 61). Our RT-PCR analysis showed a decrease in the expression of Notch-related genes after *her4.3*^+^ RGCs ablation, indicating a blockage of Notch signaling. Previous studies have shown that Notch interacts with EGFR to maintain the morphology and identity of NSCs (62), while depletion of *jagged1* has been found to decrease the number of NSCs and inhibit their self-renewal (63). Moreover, the Notch family of genes is widely expressed in the brain's vascular system and plays a crucial role in regulating angiogenesis. Specifically, the gene *jagged1* has been found to compete with *dll4* in negatively regulating angiogenesis (64). Additionally, mutations in *notch3* have been shown to affect the activation and amplification of NSCs, resulting in distinct curvature of the trunk and reduced mural cells (65, 66), This phenotype is similar to that observed after *her4.3*^+^ RGCs ablation. Based on this, we hypothesized that Notch signaling may be responsible for the observed phenotype caused by *her4.3*^+^ RGCs ablation, but we obtained a different result. The transgenic larvae, in which Notch signaling was inhibited by genetic method or chemical molecule, showed a curvature of the body but normal intersegmental vessels. There was also no significant effect on intracerebral vessels, including cell number and vessel branching. Our findings suggest that, on one hand, the curvature of the trunk caused by *her4.3*^+^ RGCs ablation may be attributed to the downregulation of Notch signaling; on the other hand, Notch signaling has different roles in regulating blood vessel development at different stages, including angiogenesis and the maintenance of basic vascular network. Our study focused on *her4.3* as a target gene in the Notch signaling. We observed a slight increase in the number of *her4.3*^+^ RGCs in the brain after inhibiting Notch signaling, which is consistent with previous research showing that Notch signaling acts as a negative regulator of RGCs, and Notch inhibition was sufficient to trigger the proliferation of RGCs and mediate the cell-cycle entry of RGCs, regardless of injury (67, 68).

Wnt signaling could promote the activation of RGCs in zebrafish. Previous studies have shown that treatment with a Wnt signaling inhibitor can suppress the proliferation and differentiation of RGCs after stab injury (48), revealing that Wnt signaling regulates the behavior of RGCs under physiological conditions. Other researchers have reported that suppressing the division of RGCs can lead to ectopic activation of Wnt signaling, resulting in vessel regression (22). However, our zebrafish model yielded opposite results. *Her4.3*^+^ RGCs ablation led to downregulation of Wnt signaling, and the disruption of Wnt signaling by using an inhibitor caused *her4.3*^+^ RGCs reduction and made the cerebral vascular network to collapse. Activation of Wnt signaling by chemical treatment did not lead to obvious blood vessel defects but partially rescued the phenotype caused by *her4.3*^+^ RGCs ablation. The discrepancy between our zebrafish model and the mouse model may be due to the different treatments used to disrupt RGCs. The cell response and signal pathway regulation were quite different after disrupting the division of RGCs or cell ablation, we still need to clarify the mechanism behind this difference. Moreover, activating Wnt signaling did not result in complete vascular regeneration, raising the possibility that Wnt signals may not be the sole key pathway involved in this process, which needs further investigation.

*Gpr124* stimulates vessel sprouting into the neural plexus and regulates brain angiogenesis dependent on the Wnt/β-catenin pathway (4). Other genes, *wnt7a*, and *wnt7b* are important for the neurogenesis of *gfap*^+^ cells, they also combine with *reck* to regulate angiogenesis and the development of BBB (53, 69). Results obtained by FISH and RT-PCR showed that *wnt7aa, wnt7bb, reck*, and *gpr124* were all significantly downregulated after *her4.3*^+^ RGCs ablation. We also used the CRISPRi strategy to specifically knock down *wnt7aa* and *wnt7bb*, and observed a similar phenotype of the collapse of the cerebral vascular network, while overexpression of *wnt7aa* or *wnt7bb* partially rescued the phenotype resulting from *her4.3*^+^ RGCs ablation, indicating that *wnt7aa* and *wnt7bb* play roles in regulating the effect of RGCs on intracerebral vessels. Notably, overexpression of *wnt7bb* appeared to be more effective than that of *wnt7aa*, possibly due to its high expression on *her4.3*^+^ RGCs and its greater significance in regulating the interaction between blood vessels and RGCs. Previous studies have shown that Wnt7a/Wnt7b play an important role in angiogenesis, and our results indicate that they also have contributions to the maintenance of vascular networks.

Apart from Notch and Wnt, other signaling pathways and factors have also been reported to play roles in the interaction between RGCs and vascular endothelial cells. VEGF is the primary pro-angiogenic growth factor described to regulate vascular development in various ways, including endothelial migration, proliferation, tube formation, and maturation (70). It has been demonstrated that RGCs secret VEGFA to induce the angiogenic response of endothelial cells, and high levels of VEGFA exert an attractive effect on invading endothelial cells from the perineural vascular plexus (17, 71). Other studies have demonstrated that VEGF induces the proliferation of glial cells and modulates vascular patterning in the spinal cord injury model (72). Another similar study on the interaction of glial cells and vascular endothelial cells revealed that *gfap*^+^ glial cells support brain vascular development through VEGF (73). As mentioned above, VEGF is an important signaling molecule that influences RGCs and blood vessels. Its precise mechanisms remain to be elucidated in the future.

TGF-β1 derived from RGCs influences angiogenesis, and inhibiting TGF-β1 disrupts vascular development, leading to reduced vascular density and branching (21). Additionally, RGCs secrete VCAM1 to regulate cortical angiogenesis at varying levels of abundance. Low-level expression of VCAM1 promoted blood vessel formation, while angiogenesis was inhibited by VCAM1 accumulation (20). It was also indicated that CXCL12- CX43 mediated the contact between microvessels and radial glial fibers, regulating neurovascular patterning (18). Certainly, the evidence related to signaling pathways that regulate interactions between RGCs and endothelial cells is still insufficient. Further studies should be conducted to clarify the molecular cues or factors essential for the interaction between RGCs and blood vessels.

In conclusion, our study demonstrates the essential role of RGCs in maintaining the cerebral vascular network, along with the regulation of Wnt signaling. After brain ischemia, RGCs were recruited to the injury site and expressed factors to rescue the injury (74, 75). Their long processes facilitated the migration of other cells to the site of injury, promoting neuronal regeneration and functional recovery (10, 76). Next, depletion of the radial glial-associated gene resulted in oxidative stress and led to age-related diseases (77). In summary, our study provides new insights into the research field of vascular development and regeneration. It also has potential implications for pathology, suggesting that RGCs may be an important desired cell type and Wnt-associated factors could be new targets.

## Experimental procedures

### Zebrafish husbandry and strains

Zebrafish strains were raised and maintained under standard laboratory conditions according to Institutional Animal Care and Use Committee protocols. The environmental conditions of the zebrafish fish room were temperature: 28.5 °C; photoperiod: 14-h light cycle/10-h dark cycle. The water quality conditions for zebrafish were pH: 7.0 to 8.0; salinity: 0.25‰; and hardness: >100 mg/L CaCO_3_. The WT zebrafish is AB strain, and the information of zebrafish strains in this study was listed in Table S1.

The zebrafish studies and experimental protocols were approved by the Animal Ethics Committee of Southwest University, Chongqing (ETHICS CODE Permit NO. IACUC-20221224-02).

### Generation of plasmids and transgenic lines

To construct the *pBluescript-hsp70l:wnt7aa; cryaa:cerulean* or *pBluescript-hsp70l:wnt7bb; cryaa:cerulean* plasmid, zebrafish *wnt7aa* and *wnt7bb* full-length coding sequences were amplified from 3 dpf cDNA. These sequences were inserted between the XmaI and NotI enzyme sites in the *pBluescript-hsp70l: cryaa:cerulean* plasmid. To construct the *hsp70l:dcas9;cryaa:cfp* plasmid, the hsp70l promoter sequence was inserted between the ApaI and XhoI enzyme sites in the *fli1a:dcas9;cryaa:cfp* plasmid to replace fli1a promoter.

*Tg(her4.3:mCherryNTR)*^*cq184*^, *Tg(her4.3:eGFPNTR)*^*cq185*^, and *Tg(her4.3:mCherryRAS)*^*cq186*^ transgenic lines were produced using the pBluescript vector. The promoter of *her4.3* is 3.4 kb and cloned from zebrafish genomics, and the following primer sequences are used for PCR amplification: F+*Apa* I: 5′- AGGGGGCCCCCTCTGTGTGAGCAGTCATGTTTGATCTC- 3′, R+*Age* I: 5′- CCCACCGGTGCCCTCTGCTGCTGATTGATCCAGTGATTG -3′. The PCR product is digested by *Apa* I and *Age* I enzymes, then ligated with eGFP-NTR, mCherry-NTR, and mCherry-RAS fragments in a *pBluescript-vector* using T4 DNA ligase. All constructs flanked by the I-SceI restriction sites were coinjected with I-SceI (NEB) into zebrafish embryos of the AB genetic background at the one-cell stage for transgenesis.

### Antibody staining

Whole-mount antibody staining in zebrafish was performed as described (78). Primary antibodies against GFP (1:500, ab6658 in Abcam and sc9996 in Santa Cruz), claudin-5 (1:500, 35–2500, Invitrogen), VE-cadherin (1:500, ab33168, Abcam), and mCherry (1:1000, ARG55723, arigo Biolaboratories) were applied. Secondary antibodies used in the study include donkey anti-goat IgG Alexa fluor 488-conjugated (1:1000, A11055, Invitrogen), donkey anti-rabbit IgG Alexa fluor 647-conjugated (1:1000, A31573, Invitrogen), donkey anti-mouse IgG Alexa fluor 647-conjugated (1:1000, A31571, Invitrogen), donkey anti-rabbit IgG Alexa fluor 568-conjugated (1:1000, A10042, Invitrogen), donkey anti-rabbit IgG Alexa fluor 488-conjugated (1:1000, A21206, Invitrogen), and donkey anti-goat IgG Alexa fluor 633-conjugated (1:1000, A21082, Invitrogen).

### Whole-mount *in situ* hybridization, combination of FISH and antibody staining

Whole-mount *in situ* hybridization, combination of FISH and antibody staining, was performed as previously described (79). Concisely, larvae were fixed in 4% paraformaldehyde in PBS at 4 °C, followed by dehydration with incubation in 100% methanol at −20 °C for at least 24 h, and detailed steps can be seen in the previous report. Primer sequences for RNA probes are produced in Table S2.

### TUNEL assay

Larvae were fixed in 4% paraformaldehyde in PBS at 4 °C overnight, followed by skin removal. TUNEL assay using the *In Situ* Cell Death Detection Kit, TMR Red (Roche) according to the manufacturer’s instruction.

### Chemical treatment

MTZ treatment was performed as previously described (28), and 5 mM MTZ (Sigma-Aldrich) dissolved in 0.2% DMSO was used for treatment for 20 h. After treatment, the larvae were washed three times with egg water and then kept in the egg water with 0.003% PTU. Since MTZ is sensitive to prolonged light exposure, larvae were protected from light upon MTZ added. As a control, larvae were incubated in egg water with 0.2% DMSO & 0.003% PTU, and the DMSO of the Control group was also removed after treatment for 20 h by changing with egg water (0.003% PTU).

For small molecule treatment, for Notch and Wnt signaling inhibition, the larvae were incubated in egg water with DAPT (50 uM, Selleck Chemicals, S1757) and XAV939 (30 uM, Sigma-Aldrich, X3004) from 3 dpf to 4 dpf. For Wnt signaling activation, after MTZ treatment at 4 dpf, the larvae were incubated in egg water with LY2090314 (2 uM, MCE, HY-16294), As a control, larvae were incubated in egg water with 0.2% DMSO & 0.003% PTU. Do not replace the new treatment solution during drug treatment.

### Heat shock

Heat shock was performed at 38.5 °C for 40 min at the indicated time frame, followed by incubation at 28.5 °C for further analysis.

### Cell sorting

The brain tissue of larvae with the *Tg(her4.3:eGFPNTR; gfap:DsRed)* double transgenic background was dissected at 9 dpf and was dissected at 4 dpf after DMSO or MTZ treatment, then cells of the brain were dissociated, and about 500, 000 cells were sorted by flow cytometry (Moflo XDP, Beckman) (78).

The brain tissue of larvae with the *Tg(her4.3:mCherryNTR; kdrl:GFP)* or *Tg(her4.3:eGFPNTR; tp1:nls-mCherry)* double transgenic background was also dissected at 4 dpf after DMSO or MTZ treatment, then cells of the brain were dissociated and about 100, 000 cells were sorted by flow cytometry for RT-PCR.

### Quantitative real-time PCR

The total RNA was extracted using the NucleoZOL (MACHEREY-NAGEL), and cDNA was synthesized using the Omniscript-Reverse Transcriptase Kit (QIAGEN). Quantitative real-time polymerase chain reaction was performed using the FastStart Universal SYBR Green Master (Roche). The relative expression levels were calculated using β-actin mRNA as a reference, and the 2^−ΔΔCt^ method was applied for Ct value analysis. Primers for RT-PCR are presented in Table S3.

### Dye injection

For PI intravital staining, 1 nl PI (100 ug/ml, MCE, HY-D0815) in DMSO was microinjected following MTZ treatment at 3.5 dpf/0.5 dpt or 4.5 dpf/1.5 dpt and incubated for 30 min at 28.5 °C, then obtain confocal images.

For the tracer experiment, 1 nl DAPI (10 mg/ml, Roche, 10236276001) was microinjected into the blood circulation system at 4 dpf/1 dpt and incubated for 30 min at 28.5 °C, then imaged by Confocal.

### Clustered regularly interspaced short palindromic repeats interference

CRISPRi was performed as described (80). At least two sgRNAs of *wnt7aa*, *wnt7ab*, *wnt7ba*, and *wnt7bb* were injected with 400 pg of *hsp70l:dcas9;cryaa:cfp* plasmid. Negative control sgRNA contained the DNA-binding element of the active gRNA but lacked the stem-loop forming region associated with binding to the dCas9 protein. SgRNAs for CRISPRi can be found at Table S4.

### Live imaging

For time-lapse live imaging, zebrafish embryos were maintained in egg water supplemented with 0.003% PTU to prevent pigmentation. Subsequently, the larvae were immobilized in 1.0% low melting point agarose and placed onto 35-mm glass bottom dishes Time-lapse images were captured using a water immersion objective mounted on the LSM880NLO (Carl Zeiss) confocal microscope equipped with a heating stage to maintain 28.5 °C, z-image stacks were collected every 10 min, and three-dimensional datasets were compiled using ZEN 2010 software (Carl Zeiss).

### Quantification and statistical analysis

All statistical data were quantified utilizing the GraphPad Prism software, while intensities and areas of the fluorescence images were measured through ImageJ. All figures, labels, arrows, scale bars, and outlines were drawn using Adobe Photoshop software.

Kaplan–Meier survival analysis was used for survival analysis. Zebrafish larvae were transferred to a 24-well plate, one per well. Survival was observed using a microscope every 24 h until 10 dpf.

Data are presented as the mean ± standard deviation. All experiments comparing treatment groups were carried out using randomly assigned siblings. After at least two repeated experiments, data were analyzed for statistical significance using two-way ANOVA by Sidak’s multiple comparisons test and two-tailed unpaired *t* test. A value of *p* < 0.05 was considered to be statistically significant. No data were excluded from analyses. The figure legends provide information on sample size (n), *p*-values for each experimental group, and statistical tests used.

## Data availability

This study did not generate/analyze dataset or code. All data generated and/or analyzed in this study are available in the article, the supplementary information, or from the corresponding author upon reasonable request. All zebrafish lines and plasmids generated in this study are available.

## Supporting information

This article contains supporting information.

## Conflict of interest

The authors declare that they have no conflict of interest with the contents of this article.
