# Supplementary material for: Her4.3+ radial glial cells maintain the brain vascular network through activation of Wnt signaling[image]
Source: J Biol Chem. 2024 Jul 15;300(8):107570. doi: 10.1016/j.jbc.2024.107570 (PMC11342778; doi:10.1016/j.jbc.2024.107570)
Supplement: Supporting Information [file mmc3.docx]

**Supporting Information**

***Her4.3*^+^ radial glial cells maintain the brain vascular network through activation of Wnt signaling**

Pengcheng Wang^1^, Lingfei Luo^1,2^, Jingying Chen^2,^*

^1^Institute of Developmental Biology and Regenerative Medicine, Southwest University, Beibei 400715, Chongqing, China

^2^ School of Life Sciences, Department of Anaesthesia of Zhongshan Hospital, Fudan University, 200438 Shanghai, China

Corresponding Author. Email: jingyingchen@fudan.edu.cn

**Material included:**

**Fig. S1 to S12;**

**Table S1 to S4;**

**Movie S1 and S2.**

**
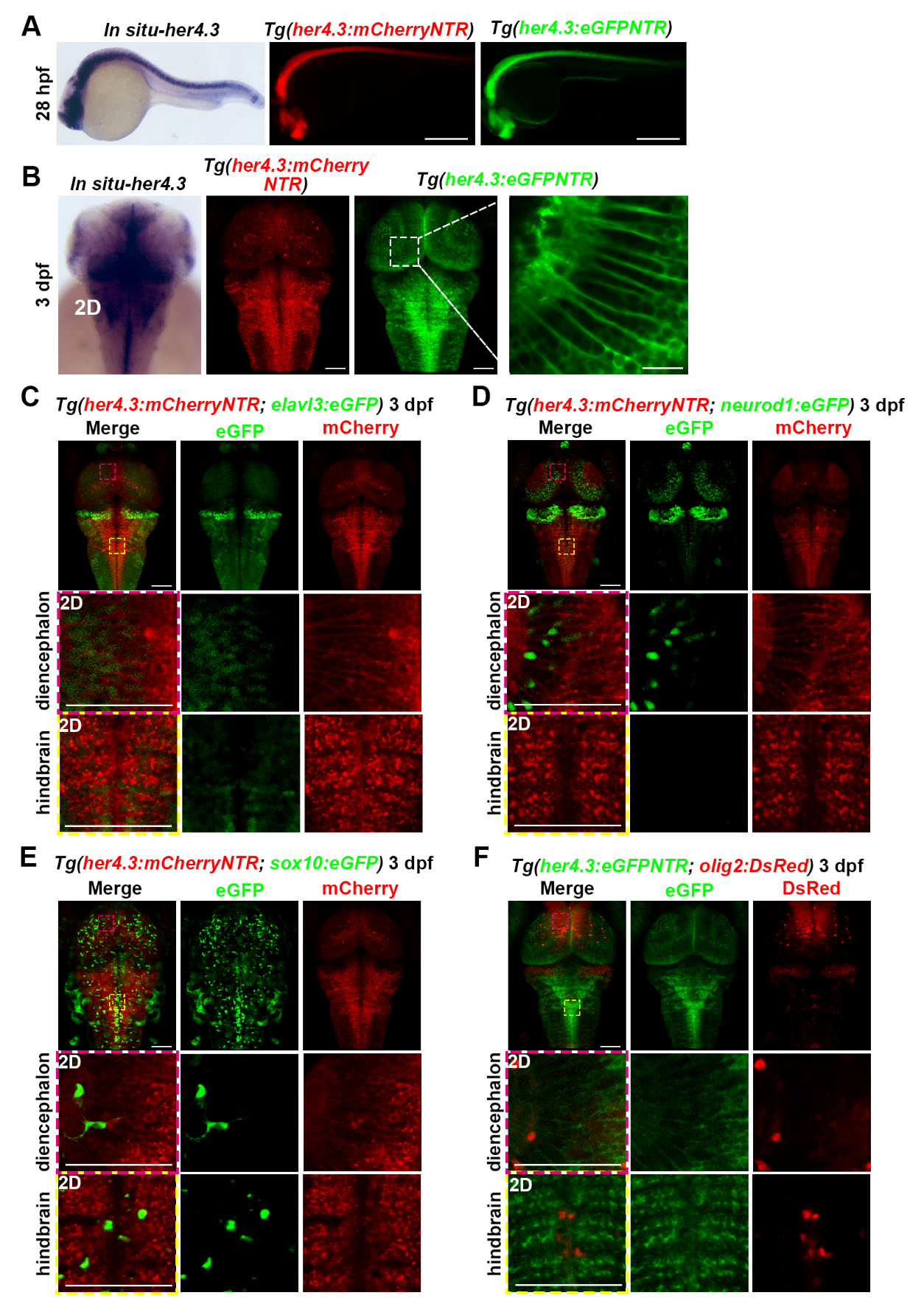
**

**Figure S1. The expression of *her4.3*** **do not overlap with other neurons or glial cells.**

(**A**) Whole-mount in situ hybridization and confocal images showed the expression of *her4.3* pattern between WT and transgenic line (n=12/12) at 28 hpf. Scale bar, 200 μm.

(**B**) In situ hybridization and confocal images at 3 dpf. n=12/12. Scale bar, 100 μm. The last column of images is a magnification of the white box in the third column of images, shown the long fiber of *her4.3*^+^ RGCs. Scale bar, 10 μm.

(**C-F**) Confocal time-lapse showed the expression pattern of *her4.3* and *elavl3* (C), *neurod1* (D), *sox10* (E), and *olig2* (F), under different double transgenic background at 3 dpf. n=12/12. The 2D image in the last two panel is a magnification of the rose or yellow box in the first image, showing *her4.3*^+^ RGCs do not overlap with other neurons or glial cells in diencephalon and hindbrain. Scale bar, 100 μm.

**
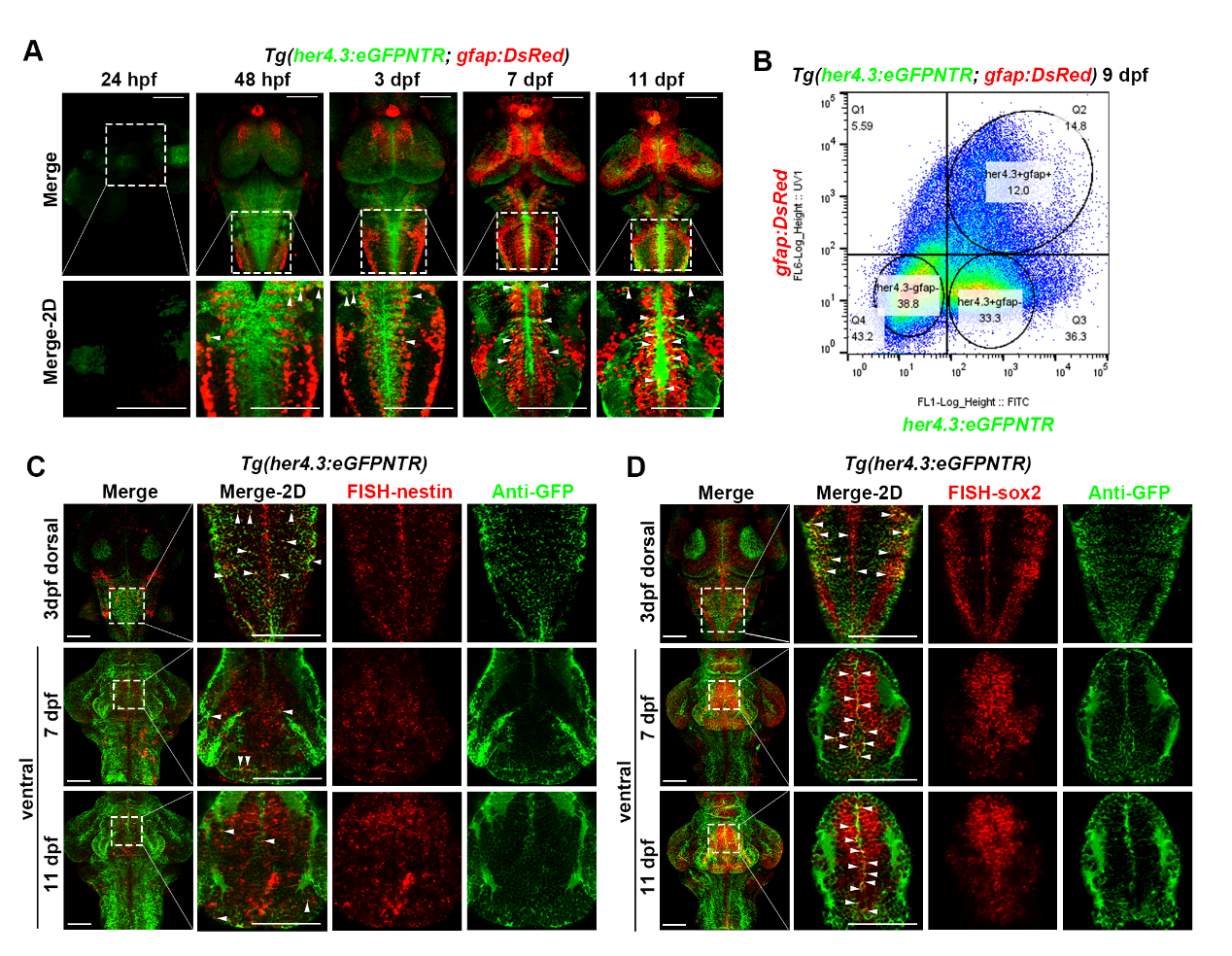
**

**Figure S2. *Her4.3* labels radial glial cells, which have some overlap regions with *gfap*, *nestin,* and *sox2*.**

(**A**) Confocal time-lapse showed the expression pattern of *her4.3* and *gfap*, at 24 hpf, 48 hpf, 3 dpf, 7 dpf, 11 dpf, under the *Tg(her4.3:eGFPNTR; gfap:DsRed)* transgenic background. The second row of 2D images is a magnification of the white box in the top images, the white arrow indicates overlap region. n=10/10.

(**B**) Flow cytometry of brain cells under the *Tg(her4.3:eGFPNTR; gfap:DsRed)* transgenic background at 9 dpf, about 500, 000 cells were sorted by flow cytometry.

(**C-D**) Fluorescent in situ hybridization (FISH) and antibody staining results showed the expression of *nestin* (n=8/8) (C) or *sox2* (n=9/10) (D) in the brain at 3 dpf, 7 dpf, and 11 dpf, under the *Tg(her4.3:eGFPNTR)* transgenic background. The second column of 2D images is a magnification of the white box in the first column of images, the white arrow indicates overlap region.

Scale bar, 100 μm.

**
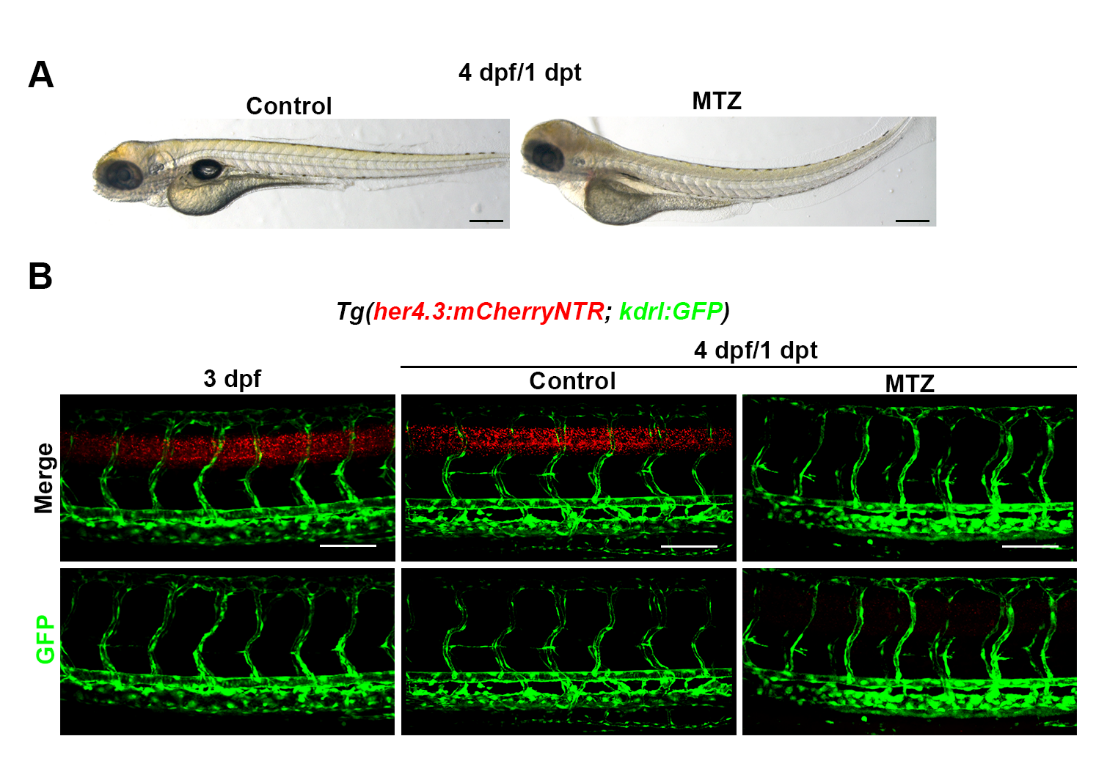
**

**Figure S3. *Her4.3*^+^ RGCs ablation has no obvious influence on blood vessels in the trunk**.

(**A**) The body shape of embryos after DMSO or MTZ treatment at 4 dpf/1 dpt. Control treated with DMSO. n=20/20. Scale bar, 200 μm.

(**B**) Confocal images showed blood vessels in the trunk, under *Tg(her4.3:mCherryNTR; kdrl:GFP)* transgenic background, after DMSO or MTZ treatment at 3 dpf and 4 dpf/1 dpt. Control treated with DMSO. n=20/20. Scale bar, 100 μm.

**
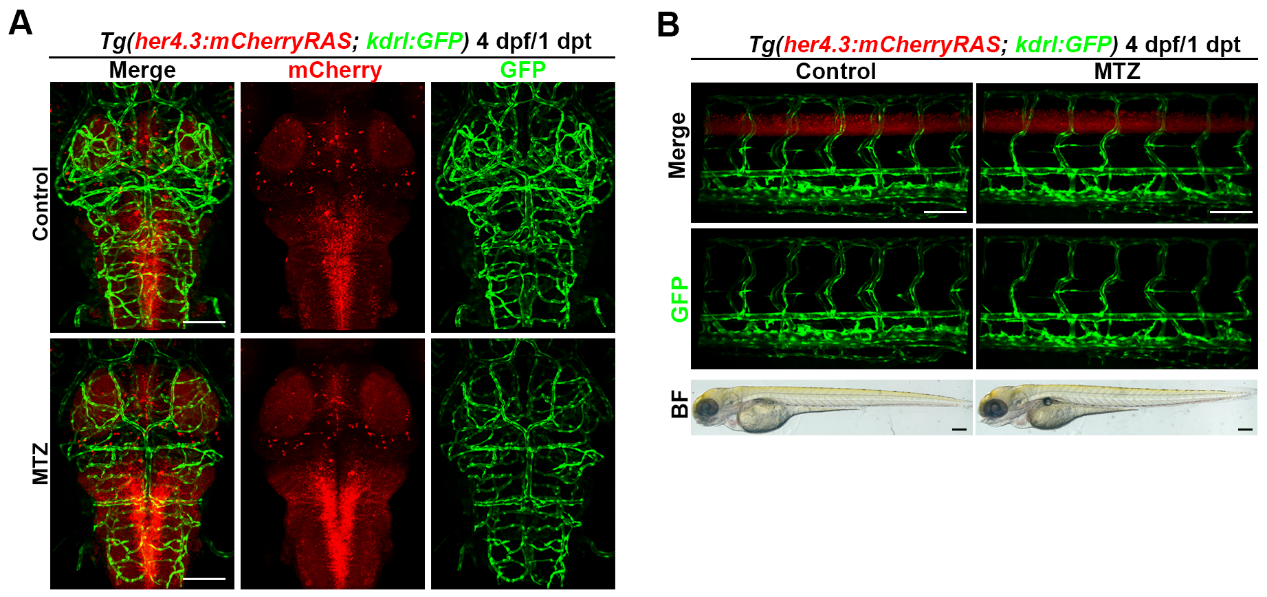
**

**Figure S4. Normal vascular morphology after MTZ treatment of non-NTR fish lines.**

(**A**) Confocal images showed cerebral vessels in *Tg(her4.3:mCherryRAS; kdrl:GFP)* after DMSO or MTZ treatment at 4 dpf/1 dpt. Control treated with DMSO. n=12/12. Scale bar, 100 μm.

(**B**) Confocal images showed blood vessels in the trunk, under *Tg(her4.3:mCherryRAS; kdrl:GFP)* transgenic background, after DMSO or MTZ treatment at 4 dpf/1 dpt. Scale bar, 100 μm. The body shape of embryos is shown in the last panel. Scale bar, 200 μm. Control treated with DMSO. n=12/12.

**
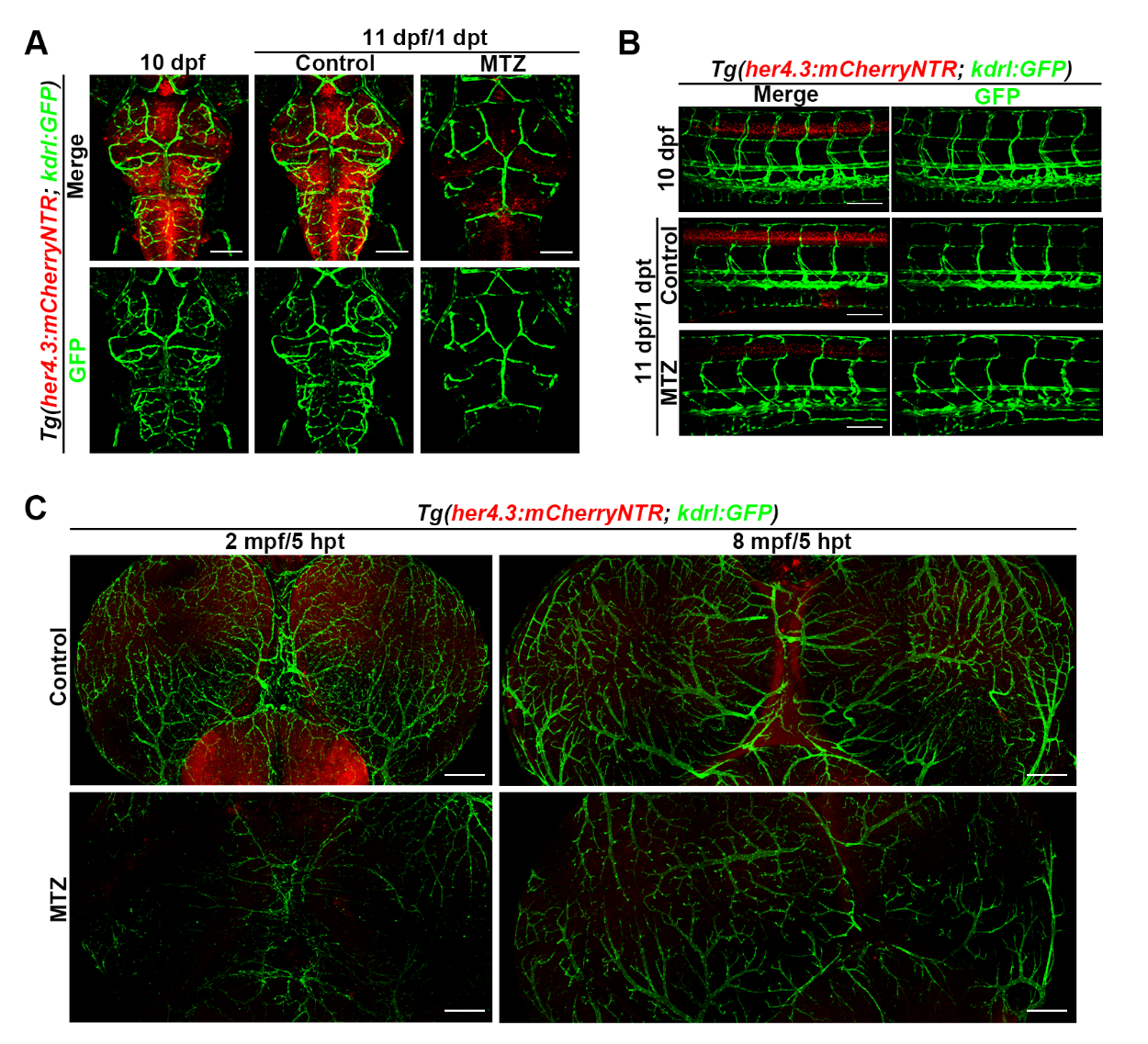
**

**Figure S5. *Her4.3*^+^ RGCs ablation destroys cerebral vessels in adult zebrafish.**

(**A**) Confocal images showed cerebral vascular disappeared from the brain after *her4.3*^+^ RGCs ablation, at 10 dpf and 11 dpf/1 dpt, under the *Tg(her4.3:mCherryNTR; kdrl:GFP)* transgenic background. Control treated with DMSO. n=12/12. Scale bar, 100 μm.

(**B**) Confocal images showed blood vessels in the trunk, under *Tg(her4.3:mCherryNTR; kdrl:GFP)* transgenic background, after DMSO or MTZ treatment at 10 dpf and 11 dpf/1 dpt. Control treated with DMSO. n=12/12. Scale bar, 100 μm.

(**C**) Confocal images showed intracerebral vessels in adult fish after DMSO or MTZ treatment at 2 mpf/5 hpt or 8 mpf/5 hpt, under *Tg(her4.3:mCherryNTR; kdrl:GFP)* transgenic background. Control treated with DMSO. n=4/4. Scale bar, 200 μm.


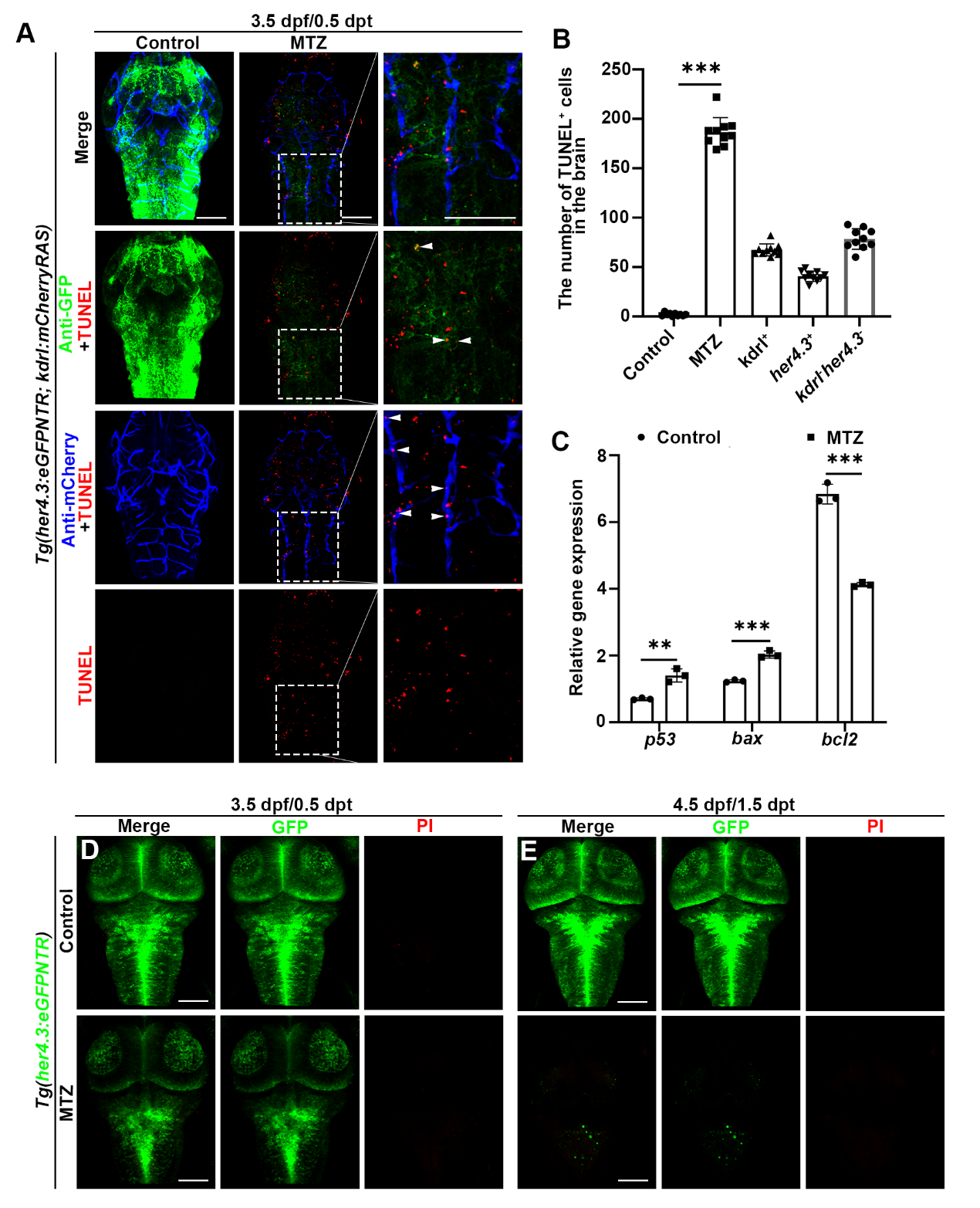


**Figure S6. MTZ treatment induces cell apoptosis, not involve cell necrosis.**

(**A**) TUNEL assay for embryos after DMSO or MTZ treatment, under the *Tg(her4.3:eGFPNTR; kdrl:mCherryRAS)* transgenic background at 3.5 dpf/0.5 dpt. Control treated with DMSO. The third column of images is a magnification of the white box in the second column of images, the white arrow indicates overlap region. n=10/12.

(**B**) Quantification of the number of TUNEL^+^ cells after DMSO or MTZ treatment, Control treated with DMSO. n=10, two-tailed unpaired t test, ***P < 0.001. Different TUNEL^+^ cells were also quantified in MTZ group, including *kdrl*^+^TUNEL^+^ cells, *her4.3*^+^TUNEL^+^ cells, and *her4.3*^-^*kdrl*^-^TUNEL^+^ cells.

(**C**) Quantitative real-time PCR data showed the relative expression level of *p53*, *bax*, and *bcl2* in the control and MTZ group at 4 dpf/1 dpt. Control treated with DMSO. n=3 technical replicates. Two-way ANOVA by Sidak’s multiple comparisons test. **P < 0.01 (p=0.003716), ***P < 0.001.

(**D** and **E**) Propidium Iodide (PI) staining for embryos in the control and MTZ group at 3.5 dpf/0.5 dpt (n=8/8) (D), or 4.5 dpf/1.5 dpt (n=8/8) (E), under the *Tg(her4.3:eGFPNTR)* transgenic background. Control treated with DMSO.

Scale bar, 100 μm. Data are represented as mean ± SD.

**
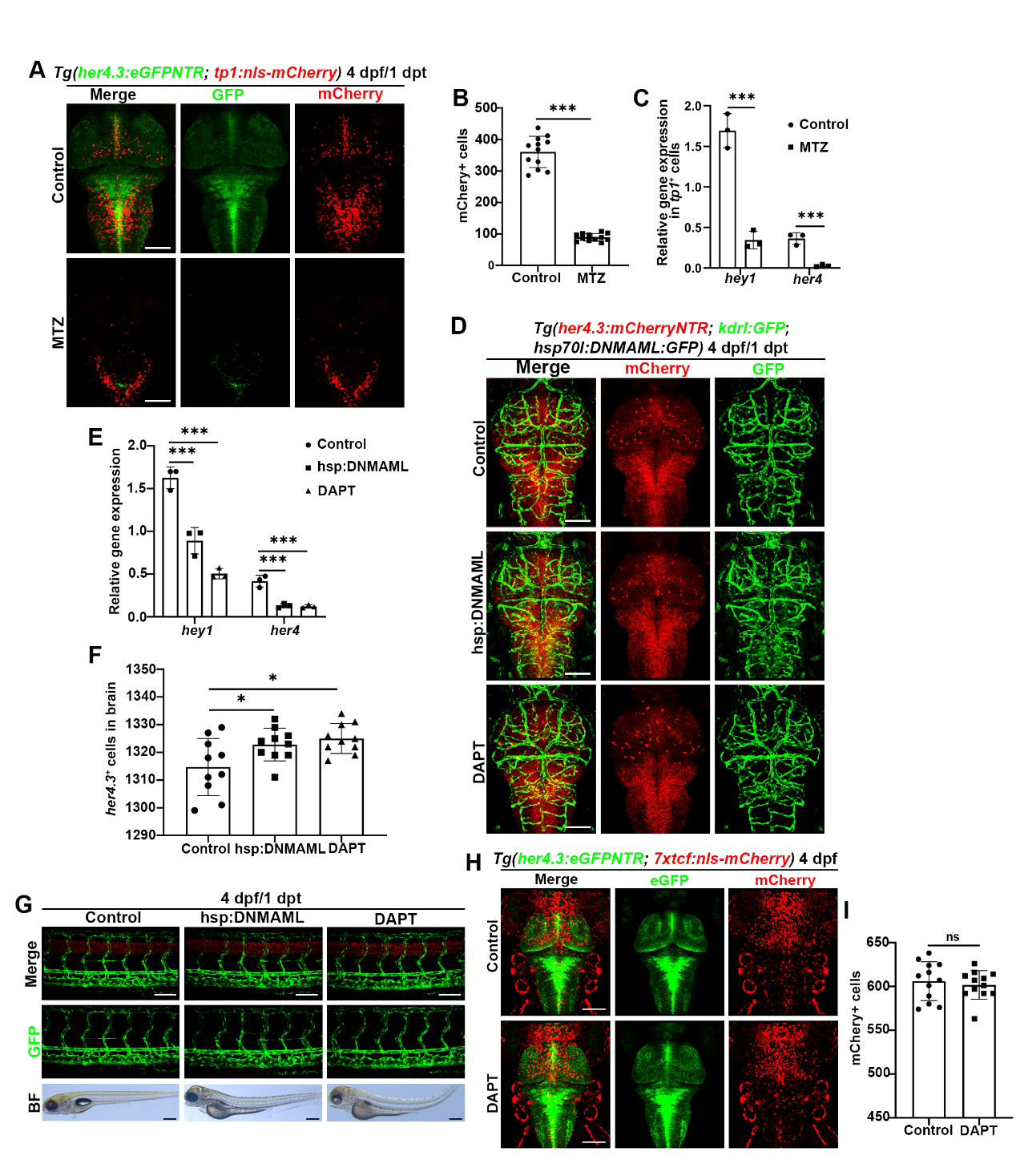
**

**Figure S7. Cerebral vascular defects** **caused by *her4.3*^+^ RGCs ablation are not mediated by Notch signaling**.

(**A**) Confocal images showed Notch signaling response to *her4.3*^+^ RGCs ablation, using Notch reporter line *Tg(tp1:nls-mCherry)* at 4 dpf/1 dpt. Control treated with DMSO. n=6/6.

(**B**) Quantification of mCherry^+^ cells after *her4.3*^+^ RGCs ablation. Control treated with DMSO. n=12, two-tailed unpaired t test, ***P < 0.001.

(**C**) Quantitative real-time PCR data showing the relative expression levels of *hey1* and *her4* in *tp1^+^* cells in control and MTZ group at 4 dpf/1 dpt. Control treated with DMSO. n=3 technical replicates. Two-way ANOVA by Sidak’s multiple comparisons test. ***P < 0.001.

(**D**) Confocal images showed embryos at 4 dpf/1 dpt after different treatments, under the *Tg(her4.3:mCherryNTR; kdrl:GFP; hsp70l:DNMAML:GFP)* transgenic background. Control treated with DMSO (n=6/6), other groups treated with DAPT (n=12/12) or heated to overexpressing DNMAML (n=11/12).

(**E**) Quantitative real-time PCR data showing the relative expression levels of *hey1* and *her4* after different treatments. Control treated with DMSO, other groups treated with DAPT or heated to overexpressing DNMAML. n=3 technical replicates. Two-way ANOVA by Sidak’s multiple comparisons test. ***P < 0.001.

(**F**) Quantification of the number of *her4.3*^+^ cells after different treatments. Control treated with DMSO, other groups treated with DAPT or heated to overexpressing DNMAML. n=10, two-tailed unpaired t test, *P < 0.5 (p=0.0448, p=0.0118).

(**G**) Confocal images showed blood vessels in the trunk or body shape at 4 dpf/1 dpt after different treatments, under the *Tg(her4.3:mCherryNTR; kdrl:GFP)* transgenic background. The body shape of embryos is shown in the last panel. Scale bar, 200 μm. Control treated with DMSO (n=6/6), other groups treated with DAPT (n=12/12) or heated to overexpressing DNMAML (n=12/12).

(**H**) Confocal images showed embryos at 4 dpf after DAPT treatment under the *Tg(her4.3:eGFPNTR; 7xtcf:nls-mCherry)* transgenic background. Control treated with DMSO (n=6/6).

(**I**) Quantification of mCherry^+^ cells after DAPT treatment. Control treated with DMSO. n=12, two-tailed unpaired t test. ns, no significance.

Scale bar, 100 μm. Data are represented as mean ± SD.


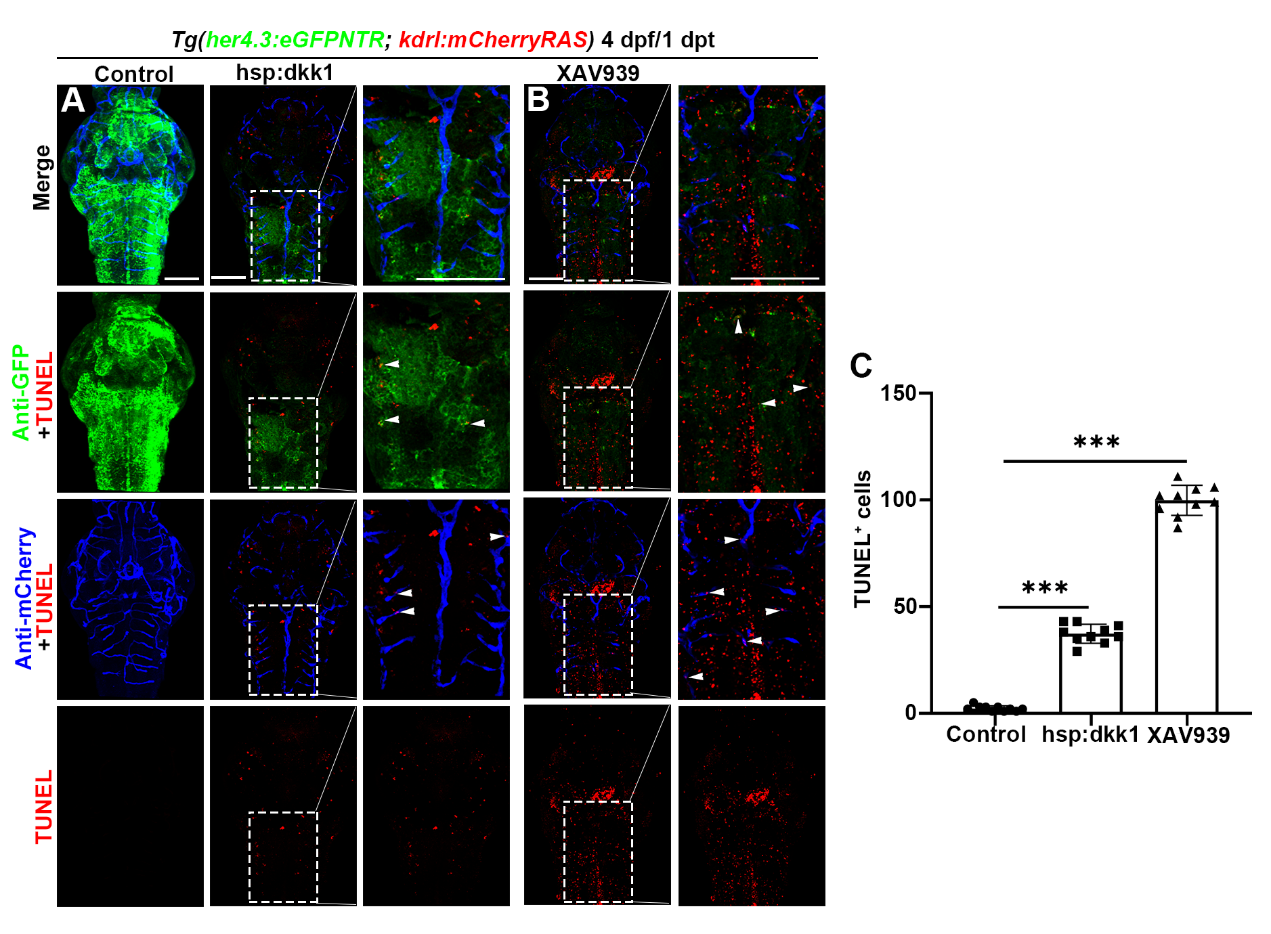


**Figure S8. Wnt signaling inhibition induces cell apoptosis.**

(**A** and **B**) TUNEL assay for embryos after different treatments. Control treated with DMSO (n=8/8), other groups heated to overexpressing dkk1 (n=9/12) (A), or treated with XAV939 (n=12/12) (B). The last column of images in (A) or (B) is a magnification of the white box in the preceding column images, the white arrow indicates the overlap region. Scale bar,100 μm.

(**C**) Quantification of the number of TUNEL^+^ cells after different treatments. Control treated with DMSO, other groups treated with XAV939, or heated to overexpressing dkk1. n=10, two-tailed unpaired t test, ***P < 0.001.

**
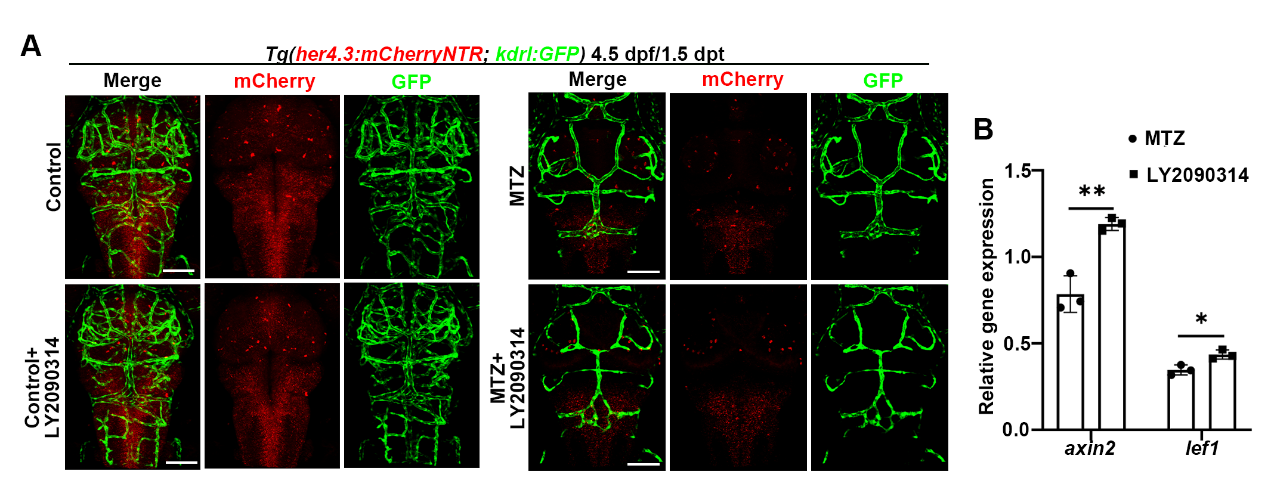
Figure S9. The phenotype of larvae treated with Wnt activator after *her4.3*^+^ RGCs ablation.**

(**A**) Confocal images showed embryos at 4.5 dpf/1.5 dpt after different treatments, under the *Tg(her4.3:mCherryNTR; kdrl:GFP)* transgenic background. In the picture on the left, the upper group was treated with DMSO, and the bottom group was treated with DMSO + LY2090314, n=10/10. Other groups treated with MTZ or MTZ+LY2090314. n=8/8. Scale bar, 100 μm.

(**B**) Quantitative real-time PCR data showing the relative expression levels of *axin2* and *lef1* in MTZ and LY2090314 group at 5.5 dpf/2.5 dpt. n=3 technical replicates. Two-way ANOVA by Sidak’s multiple comparisons test. *P < 0.5 (p=0.017551), **P < 0.01(p=0.003320).

**
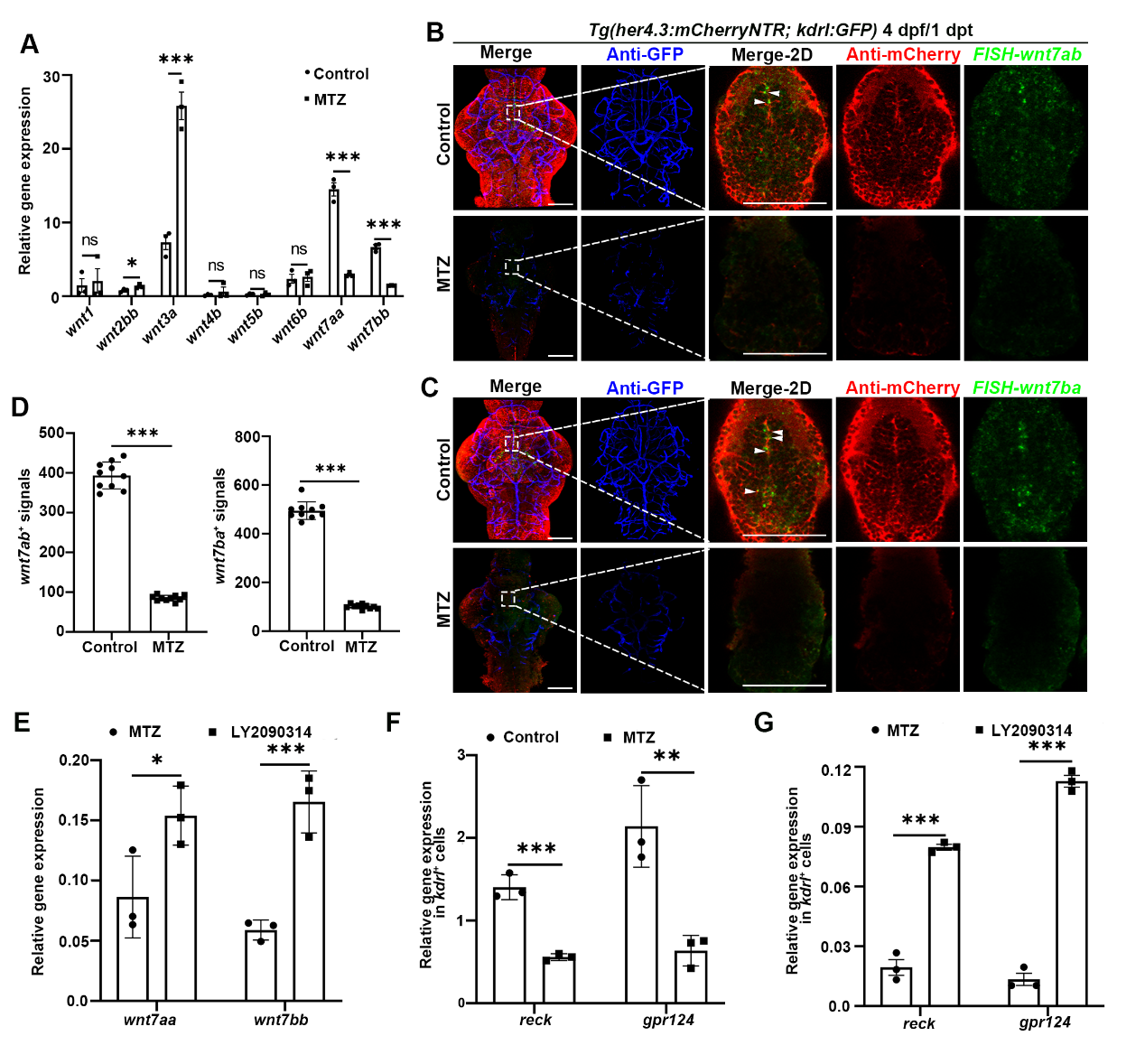
**

**Figure S10. Wnt ligands and receptors were downregulated after *her4.3*^+^ RGCs ablation.**

(**A**) Quantitative real-time PCR data showing the relative expression levels of target genes associated with Wnt in the control and MTZ group at 4 dpf/1 dpt. Control treated with DMSO. n=3 technical replicates. Two-way ANOVA by Sidak’s multiple comparisons test, ns, no significance, *P < 0.05 (p=0.041958), ***P < 0.001.

(**B** and **C**) Fluorescent in situ hybridization (FISH) and antibody staining results showed the expression of *wnt7ab* (n=12/12) (B) or *wnt7ba* (n=12/12) (C) after DMSO or MTZ treatment at 4 dpf/1 dpt, under the *Tg(her4.3:mCherryNTR; kdrl:GFP)* transgenic background. Control treated with DMSO. Scale bar, 100 μm. Higher magnification 2D images of the white framed areas are displayed, the white arrow indicates the overlap region. Scale bar, 50 μm.

(**D**) Quantification of the number of *wnt7ab* and *wnt7ba* signals after DMSO or MTZ treatment. Control treated with DMSO. n=10. two-tailed unpaired t test, ***P < 0.001.

(**E**) Quantitative real-time PCR data showing the relative expression levels of *wnt7aa* and *wnt7bb* in MTZ and LY2090314 group at 5.5 dpf/2.5 dpt. n=3 technical replicates. Two-way ANOVA by Sidak’s multiple comparisons test. *P < 0.5 (p=0.049030), ***P < 0.001.

(**F**) Quantitative real-time PCR data showing the relative expression levels of *reck* and *gpr124* in *kdrl^+^* endothelial cells in control and MTZ group at 4 dpf/1 dpt. Control treated with DMSO. n=3 technical replicates. Two-way ANOVA by Sidak’s multiple comparisons test. **P < 0.01 (p=0.007779), ***P < 0.001.

(**G**) Quantitative real-time PCR data showing the relative expression levels of *reck* and *gpr124* in *kdrl^+^* endothelial cells in MTZ and LY2090314 group at 5.5 dpf/2.5 dpt. n=3 technical replicates. Two-way ANOVA by Sidak’s multiple comparisons test. ***P < 0.001.

Data are represented as mean ± SD.

**
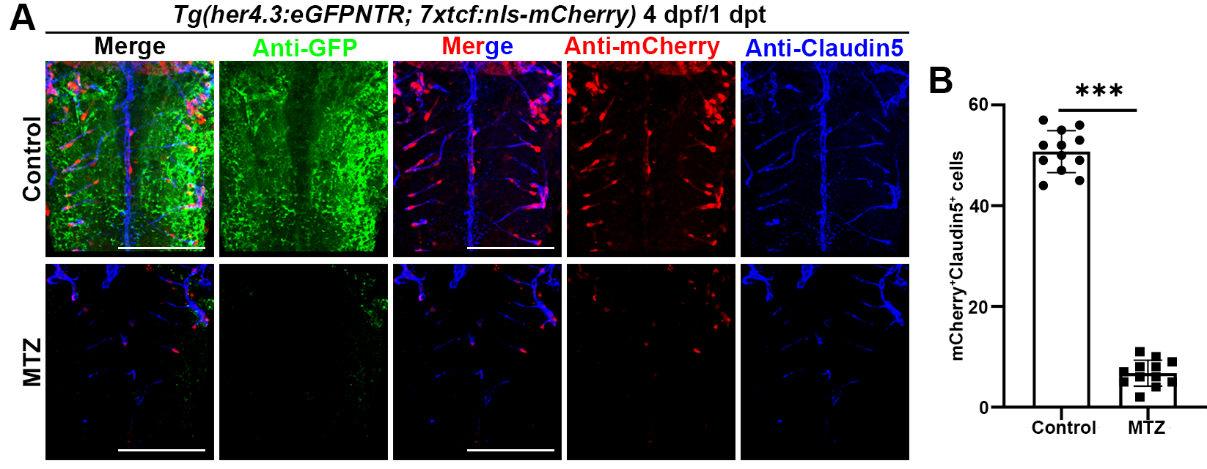
Figure S11 Wnt signaling was downregulated in endothelial cells.**

(**A**) Antibody staining for Claudin5 (n=12/12) under the *Tg(her4.3:eGFPNTR; 7xtcf:nls-mCherry)* transgenic background after different treatment at 4 dpf/1 dpt. Control treated with DMSO. Scale bar, 100 μm.

(**B**) Quantification of the number of mCherry^+^Claudin5^+^ cells after *her4.3*^+^ RGCs ablation. Control treated with DMSO. n=12. Data are represented as mean ± SD. two-tailed unpaired t test, ***P < 0.001.

**
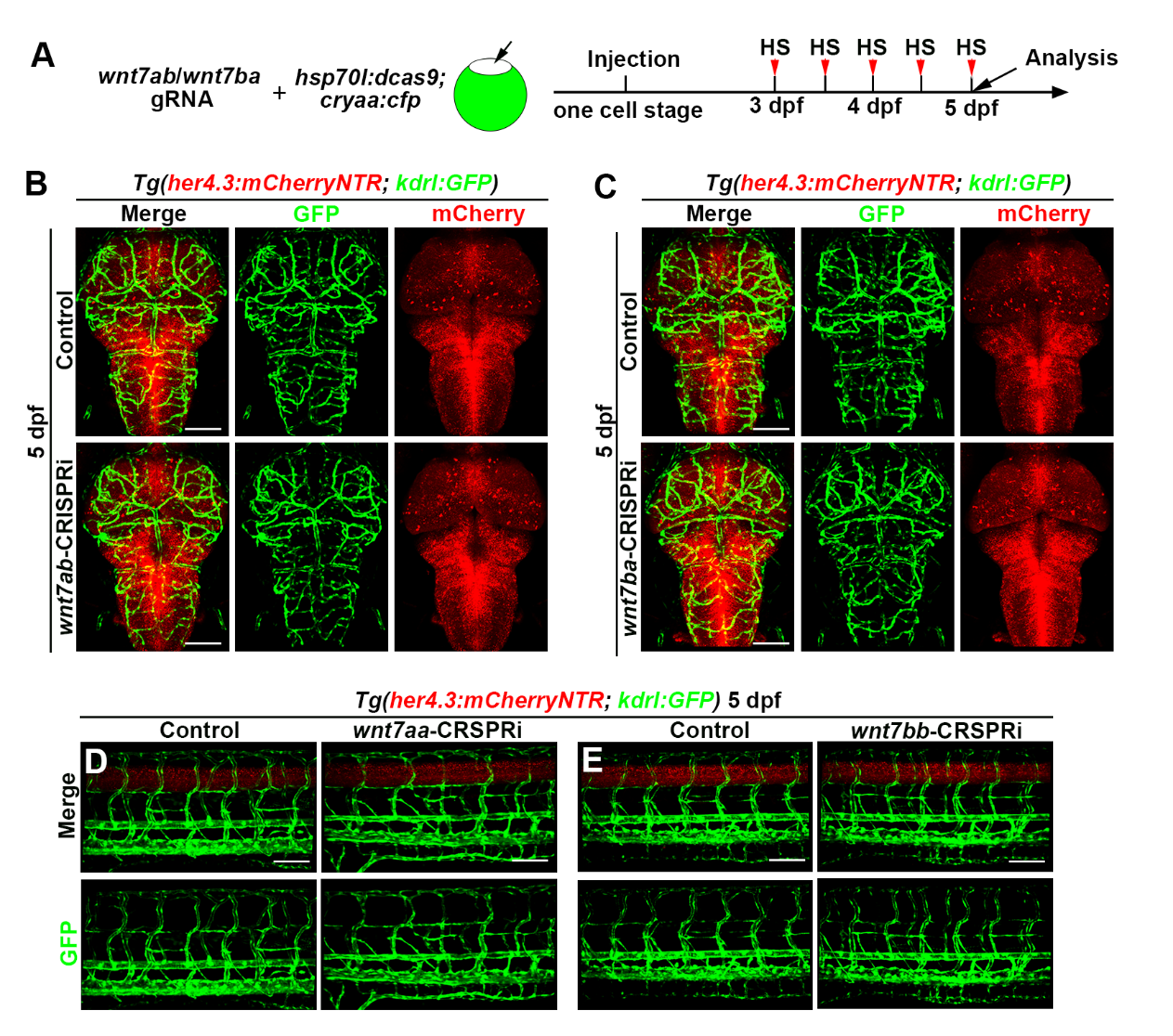
**

**Figure S12. *Wnt7ab* and *wnt7ba* downregulation cannot phenocopy the disruption of the cerebrovascular network caused by *her4.3*^+^ RGCs ablation.**

(**A**) Overview of the knockdown strategy for *wnt7ab* or *wnt7ba* and the timepoint of heat-shock (red arrow).

(**B** and **C**) Confocal images showed the cerebral vascular pattern after heat-shock at 5 dpf, under the *Tg(her4.3:mCherryNTR; kdrl:GFP)* transgenic background. *wnt7ab*-CRISPRi (n=10/10) (B) and *wnt7ba*-CRISPRi (n=10/10) (C). Control treated with DMSO & heat shock (n=6/6).

(**D** and **E**) Confocal images showed blood vessels in the trunk are normal after CRISPRi at 5 dpf, *under the Tg(her4.3:mCherryNTR; kdrl:GFP) transgenic background. wnt7aa*-CRISPRi (D) and *wnt7bb*-CRISPRi (E).

Scale bar, 100 μm.

**Table S1. Zebrafish strains**

| **Zebrafish Strain** | **Identifier** | **Experiment** |
| --- | --- | --- |
| *WT-AB* | N/A | ALL Figures |
| *Tg(her4.3:mCherryNTR)^cq184^* | N/A | ALL Figures |
| *Tg(her4.3:eGFPNTR)^cq185^* | N/A | ALL Figures |
| *Tg(kdrl:GFP)* | ZFIN: ZDB-TGCONSTRCT-070117-47 | ALL Figures |
| *Tg(kdrl:mCherryRAS)* | N/A | ALL Figures |
| *Tg(her4.3:mCherryRAS)^cq186^* | N/A | Fig S4 |
| *Tg(gfap:DsRed)* | N/A | Fig S2 |
| *Tg(pdgfrb:GFP)* | ZFIN: ZDB-ALT-160609-1 | Fig 2 |
| *Tg(acta2:GFP)* | ZFIN: ZDB-TGCONSTRCT-120726-1 | Fig 2 |
| *Tg(tp1:nls-mCherry)* | ZFIN: ZDB-TGCONSTRCT-090625-1 | Fig S7 |
| *Tg(7xtcf:nls-mCherry)* | ZFIN: ZDB-ALT-181004-2 | Fig 4, Fig S11 |
| *Tg(hsp:dkk1)* | N/A | Fig 4, Fig S8 |
| *Tg(olig2:DsRed)* | ZFIN: ZDB-TGCONSTRCT-080321-1 | Fig S1 |
| *Tg(elval3:GFP)* | ZFIN: ZDB-ALT-211018-1 | Fig S1 |
| *Tg(neurod1:GFP)* | N/A | Fig S1 |
| *Tg(sox10:eGFP)* | N/A | Fig S1 |
| *Tg(gata1a:Dsred)* | ZFIN:ZDB-TGCONSTRCT-070117-38 | Movie S1 |

**Table S2. Primer sequences for RNA probe**

| ***sox2*-F** | GTAGACTTTCGAGAAAATCGG |
| --- | --- |
| ***sox2*-R** | CATTTTACATATGCGATAAGG |
| ***nestin*-F** | GATCTCAGCAAGAGCAACTGG |
| ***nestin*-R** | CTCATCACCAGATGACCAGG |
| ***wnt7aa*-F** | ATGAGCAGGAAAACGCGCCG |
| ***wnt7aa*-R** | TCACTTGCATGTGTACACTTC |
| ***wnt7ab*-F** | ATGGGCATCAACGAGTGCCA |
| ***wnt7ab*-R** | TCATTTACAGGTGTACACCTC |
| ***wnt7ba*-F** | CAGCTGCTTACATCAGTGTG |
| ***wnt7ba*-R** | TCATTTACAAGTGAACACCTC |
| ***wnt7bb*-F** | ATGGTCAGACCCCTACGCTTG |
| ***wnt7bb*-R** | TCATTTGCAGGTAAACACCTC |
| ***her4.3-F*** | ATGACTCCTACAATCACTGG |
| ***her4.3-R*** | CCAGGGTCTCCAGATGTGAC |

**Table S3. Primer sequences for RT-PCR**

| ***β-actin*-F** | CGTCTGGATCTAGCTGGTCGTGA |
| --- | --- |
| ***β-actin*-R** | CAATTTCTCTTTCGGCTGTGGTG |
| ***hey1*-F** | GAGGGACCGGATAAATAACAG |
| ***hey1*-R** | TCTAGTTTAGCTGAGCCCTG |
| ***her4*-F** | AGGAGAACTGAACACAAGACAC |
| ***her4*-R** | TGCTGTTGATTCGCTCTCG |
| ***axin2*-F** | CAATGGACGAAAGGAAAGATCC |
| ***axin2*-R** | AGAAGTACGTGACTACCGTC |
| ***lef1*-F** | CAGACATTCCCAATTTCTATCC |
| ***lef1*-R** | TGTGATGTGAGAACCAACC |
| ***cyclind1*-F** | AGGCTTTTGAAACGTAAGCCTGCGG |
| ***cyclind1*-R** | AGGTACACTTGGGCATCCGTGCA |
| ***cldn5a*-F** | GCAGTGCACAAACTGCATCA |
| ***cldn5a*-R** | GGTTATAGAAGTCGGAGATG |
| ***mfsd2a*-F** | CTCTTCACTTCGCTAGCCTTCATG |
| ***mfsd2a*-R** | CGATGTAAACAGCAGTCTTTTTCCC |
| ***VE-cadherin*-F** | CTGCCACTACCGTTGTTACC |
| ***VE-cadherin*-R** | TGTAGCGCAAATGTTGGATC |
| ***p53*-F** | CCCGGATGGAGATAACTTG |
| ***p53*-R** | CACAGTTGTCCATTCAGCAC |
| ***bax*-F** | CAGGGTGGATGGGACGGAAT |
| ***bax*-R** | TCACCCTCTGTTCACCGTCT |
| ***bcl-2*-F** | AAATGGAGGTTGGGATGCCTT |
| ***bcl-2*-R** | CGATGGTCACTCCTGCCAAG |
| ***wnt7aa-F*** | AGTAAGGAGGCAGCCTTCAC |
| ***wnt7aa-R*** | CTGCTTGATCTCCCGTGCATC |
| ***wnt7bb-F*** | CATCATCGTGATCGGAGAAG |
| ***wnt7bb-R*** | GTGATGGCGTAGGTGAATGC |
| ***wnt1-F*** | TCGTTGGAACTGTCCCACTAC |
| ***wnt1-R*** | TAGTCACAGGTGCAGGACTCAAT |
| ***wnt2bb-F*** | GTGGCGCTAAGGAGTGGATT |
| ***wnt2bb-R*** | CTCTCCTCGTTCGTCACTGG |
| ***wnt3a-F*** | CACCTCCCATCCCTTCCTA |
| ***wnt3a-R*** | CCGTTCTGCTCAAGTGTCCT |
| ***wnt4b-F*** | GGAGCCTTTCGGACCTCAAA |
| ***wnt4b-R*** | GGACAGACGTGCCAGAGAAA |
| ***wnt5b-F*** | ATGCTAGCAGAGACACTATTACTGG |
| ***wnt5b-R*** | TTTTTGGAGTGAAATGGTAAGTGTT |
| ***wnt6b-F*** | GCGTTGTGCATTACCTGACC |
| ***wnt6b-R*** | CGAAGGCATTTCGTGATCGT |
| ***gpr124-F*** | CCTGATGCAGGTAGATGATC |
| ***gpr124-R*** | CCGTGCAGCTAATGCCAGTG |
| ***reck-F*** | GCAAAGAGCTCAGCTACTGC |
| ***reck-R*** | GCTACAGTCTTCCACATCTC |

**Table S4. SgRNAs for CRISPRi**

| ***wnt7aa*-gRNA 1** | TTCAGCCTGTGCTTTGTTAA |
| --- | --- |
| ***wnt7aa*-gRNA 2** | TGAGCAGGAAAACGCGCCGC |
| ***wnt7ab*-gRNA 1** | GACGGTCCTCTCTCCGAGCG |
| ***wnt7ab*-gRNA 2** | GGCCGTGCTTGAACTGAAAT |
| ***wnt7ba*-gRNA 1** | GGCAGATGGCACGCTGTCGG |
| ***wnt7ba*-gRNA 2** | CGACTGAAGACAAAGCCCTT |
| ***wnt7bb*-gRNA 1** | GCGCGACGAGAAGAGGATCA |
| ***wnt7bb*-gRNA 2** | CGATCCGACGCCTTTTCTGA |

**
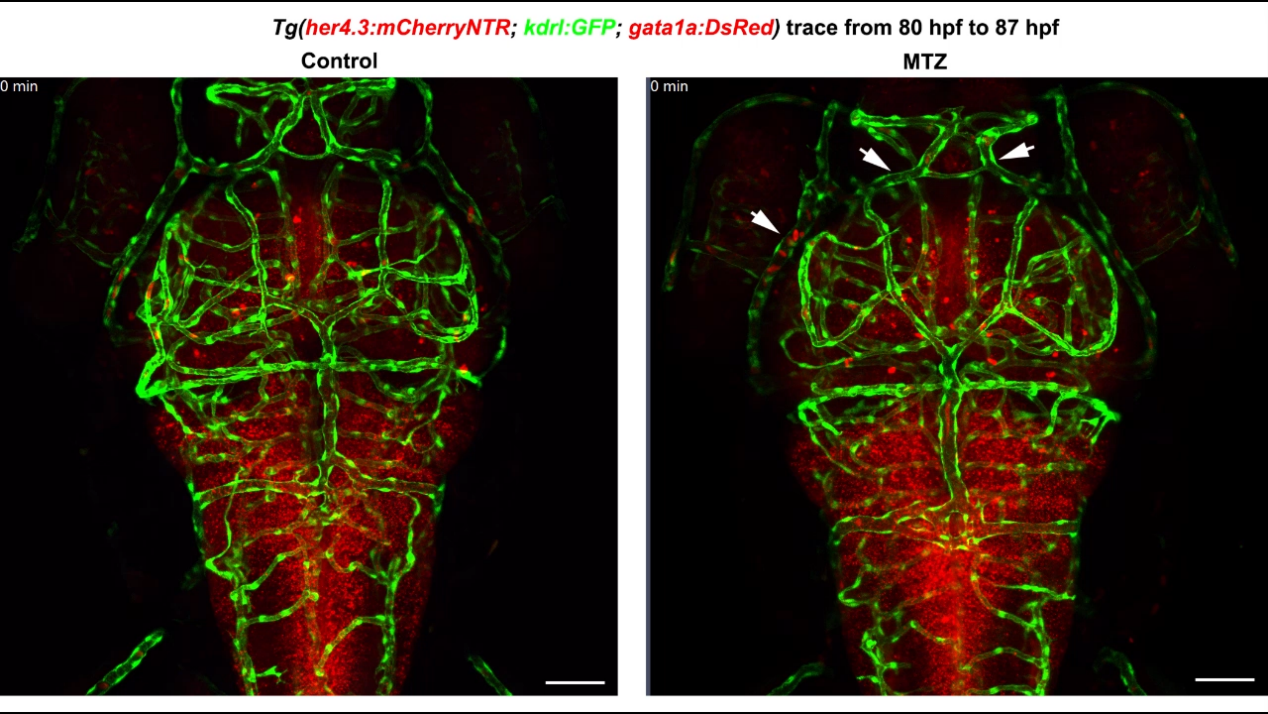
**

**Movie S1. The dynamic changes of intracerebral vessels and blood cells after *her4.3*^+^ RGCs ablation.**

The *Tg(her4.3:mCherryNTR; kdrl:GFP;gata1a:Dsred)* transgenic line was used for the time-lapse image after DMSO or MTZ treatment from 80 hfp to 87 hpf. Control treated with DMSO. The *kdrl*^+^ intracerebral vessels disappeared from the brain with the reduction of *her4.3*^+^ RGCs after MTZ treatment, whereas blood vessels on the cranial head were still present and *gata1a*^+^ blood cells could be observed flowing through the vessels. Arrowheads indicate the flow of blood cells through the blood vessels. Scale bar, 100 μm.


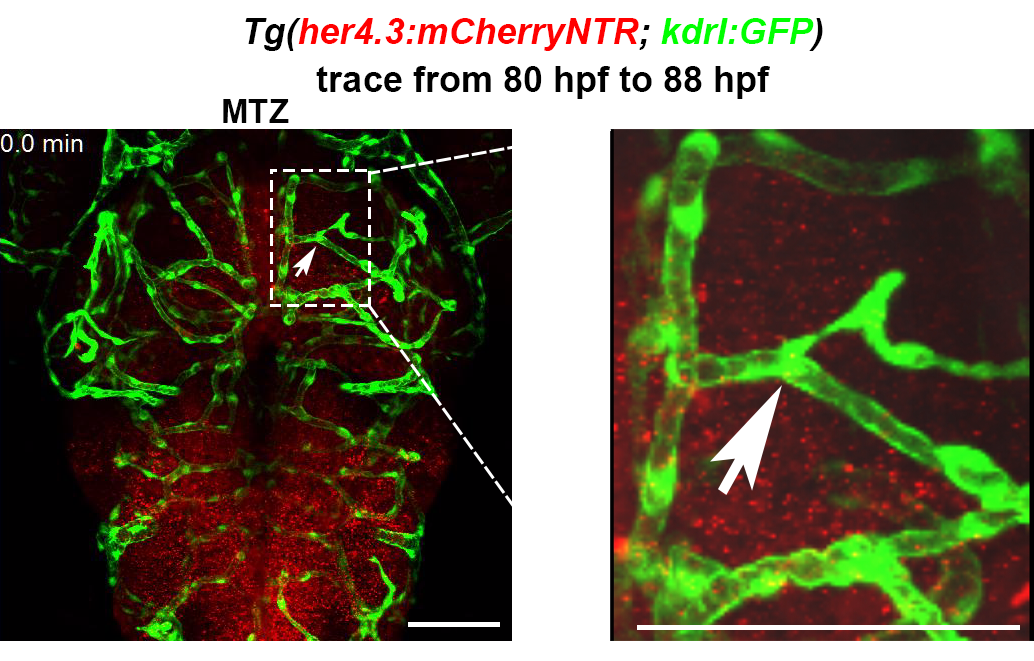
**Movie S2. The dynamic changes of intracerebral vessels and her4.3+ RGCs after MTZ treatment**

The *Tg(her4.3:mCherryNTR; kdrl:GFP)* transgenic line was used for the time-lapse image after MTZ treatment from 80 hfp to 88 hpf. The *kdrl*^+^ intracerebral vessels disappeared from the brain with the reduction of *her4.3*^+^ RGCs after MTZ treatment. The right panel is a magnification of the white box in the left panel. Arrowheads indicate single vessels. Scale bar, 100 μm.
